# Multiple *Doris* “*kerguelenensis*” (Nudibranchia) species span the Antarctic Polar Front

**DOI:** 10.1002/ece3.9333

**Published:** 2022-09-20

**Authors:** Paige J. Maroni, Nerida G. Wilson

**Affiliations:** ^1^ School of Biological Sciences (M092) University of Western Australia Crawley Western Australia Australia; ^2^ Western Australian Museum, Research & Collections Welshpool Western Australia Australia; ^3^ Securing Antarctica's Environmental Future Western Australian Museum Welshpool Western Australia Australia

**Keywords:** Antarctica, cytochrome oxidase I, direct development, mtDNA, phylogeography

## Abstract

Despite strong historical biogeographical links between benthic faunal assemblages of the Magellan region of South America and the Antarctic Peninsula, very few studies have documented contemporary movement and gene flow in or out of the Southern Ocean, especially across the Antarctic Polar Front (APF). In fact, oceanographic barriers such as the APF and Antarctica's long geologic isolation have substantially separated the continents and facilitated the evolution of endemic marine taxa found within the Antarctic region. The Southern Ocean benthic sea slug complex, *Doris* “*kerguelenensis*,” are a group of direct‐developing, simultaneous hermaphrodites that lack a dispersive larval stage. To date, there are 59 highly divergent species known within this complex. Here, we provide evidence to show intraspecific genetic connectivity occurs across the APF for multiple species within the *D.* “*kerguelenensis*” nudibranch species complex. We addressed questions of genetic connectivity by examining the phylogeographic structure of the three best‐sampled *D.* “*kerguelenensis*” species and another three trans‐APF species using the protein coding mtDNA gene, cytochrome oxidase I. We also highlight alternative refugia uses among species with the same life history traits (i.e., benthic and direct developers) and for some species, extremely large distributions are established (e.g., circumpolarity). By improving our sampling of these nudibranchs, we gain better insight into the population structure and connectivity of the Antarctic region. This work also demonstrates how difficult it is to make generalizations across Antarctic marine species, even among ecologically‐similar, closely related species.

## Graphical abstract

Here, we provide evidence to show genetic connectivity occurs across the Antarctic Polar Front (APF) within multiple species from the *Doris* “*kerguelenensis*” sea slug species complex. This appears to be the first example of a benthic direct‐developing species that spans the APF from the southern South America continental shelf to the Antarctic continental shelf. Our work demonstrates the value of increasing geographic scope in sampling and highlights how difficult it is to make generalizations about Antarctic species, even among closely related species.

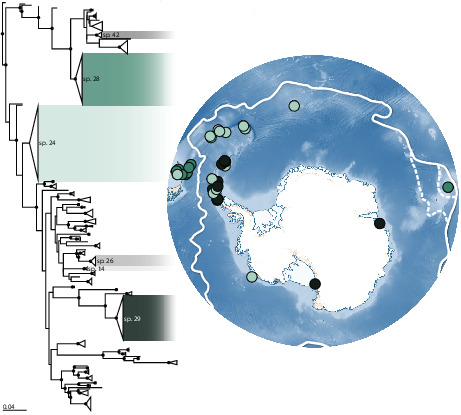

## 1 INTRODUCTION

**FIGURE 1:**
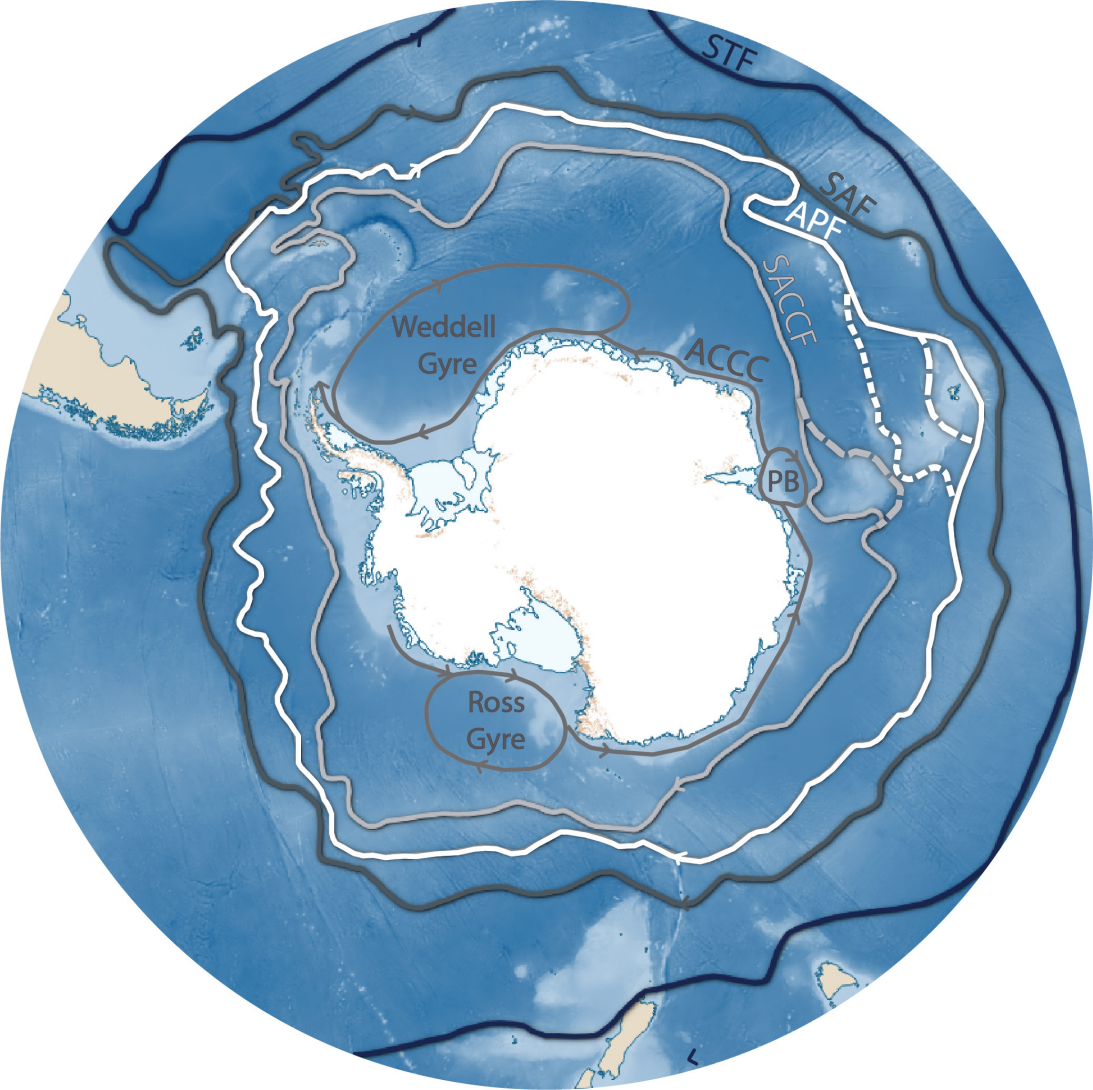
Map of Antarctica, Southern Ocean (SO) bathymetry, major fronts, currents, and gyres. Sub‐Tropical Front (STF) (navy), Sub‐Antarctic Front (SAF) (dark gray), Antarctic Polar Front (APF) (white), Southern Antarctic Circumpolar Current Front (SACCF) (light gray), Antarctic Count Current Front (ACCC) (or Antarctic Slope Front) (medium gray). New additions to APF (north to south, dashed lines) Park et al. (2014) and Sokolov and Rintoul (2009) for the SACCF. Base map and original fronts (solid lines) retrieved from Quantarctica 3.2 (Matsuoka et al., 2021). Gyre information: Weddell Gyre (Vernet et al., 2019), Ross Gyre (Roach & Speer, 2019), Prydz Bay Gyre (PB) (Nunes Vaz & Lennon, 1996; Williams et al., 2016).

Antarctica's long geologic and oceanographic isolation (approx. 25–35 million years, Barker & Burrell, 1977; Barker et al., 2007; Livermore et al., 2004; Pfuhl & McCave, 2005) has been critical in fuelling the diversity of endemic marine taxa within this region (Arntz et al., 1997; Linse et al., 2006). Such high levels of faunal endemism have been discussed among many Antarctic lineages of fish (e.g., Bargelloni et al., 2000; Clarke & Johnston, 2003), and marine invertebrate groups such as amphipods, isopods, mollusks, pycnogonids, and ophiuroids (see: Brandt, 1999; Leese et al., 2010; Linse et al., 2006; Martín‐Ledo & López‐González, 2014; Munilla & Membrives, 2009). The disintegration of the once continuous faunal assemblage between southern South America and Antarctica (i.e., the opening of the Drake Passage ~35 Mya, Barker & Burrell, 1977) resulted in the onset of the Antarctic Circumpolar Current (ACC) (Box 1; Figure 1), global cooling and subsequent Antarctic glaciations (Kennett, 1977; Kennett et al., 1975), which was critical in promoting such high levels of polar diversity. Throughout glacial maxima, it has been suggested that benthic fauna either persisted within isolated, ice‐free continental shelf refugia (Clarke & Crame, 1989, 1992; Pearse et al., 2009), or migrated to and persisted in the surrounding deep‐sea regions or on sub‐Antarctic/Antarctic islands (Lau et al., 2020; Provan & Bennett, 2008; Thatje et al., 2005). In response to physical (Clarke & Crame, 1989, 1992), ecological (Chown et al., 2015; Convey et al., 2009, 2014), and physiological (Harper et al., 2012; Lau et al., 2021) evolutionary drivers, selection upon Antarctic benthic fauna has favored eurybathy (i.e., capacity to span a large depth range), long life cycles, slow growth rates/slow metabolism, and direct development (Allcock & Strugnell, 2012; Thatje, 2012).

BOX 1 **The interaction between the Southern Ocean, the Antarctic seas and the benthos**

**Defining the Southern Ocean**

The Southern Ocean is unique as an oceanographic environment, global climate regulator, an important ecoregion housing a diverse range of endemic flora and fauna and is among one of the most data sparse regions throughout all major ocean basins (Chapman et al., 2020). The Southern Ocean, or rather, the concept of the Southern Ocean (collection of seas around Antarctica) is an extreme environment in many of these respects and is generally defined as bounded between the Antarctic continent and the Sub‐Tropical Front (STF) (see: Stark et al., 2019). The Southern Ocean and where its boundaries lie has been variously defined throughout literature with examples extending the boundary from “the parallel of 60°S to the north and the Antarctic continent to the south” (IHO, 2002) to “the seasonally fluctuating natural boundary of the Antarctic Convergence” (Pyne, 2017).

**Important Southern Ocean current systems**

The ACC, today is defined as the largest ocean current (23,000 km), powered almost entirely by wind, and extending to the seabed (2000–4000 m), where its path is determined by topography (Lazarus & Caulet, 1993). Traditionally, three primary oceanic fronts make up the ACC; the Sub‐Antarctic Front (SAF), the APF, and the Southern Antarctic Circumpolar Current Front (SACCF) (north to south; Figure 1). To the north of these fronts is the STF (Figure 1) (between 35°S and 45°S), which separates the waters of the Southern Ocean from saltier, warmer, subtropical waters to the north (Klinck & Nowlin, 2001). To the south, there is a fifth frontal zone, between the ACC and the Antarctic continent called the westward flowing Antarctic Coastal Counter Current (ACCC), or Antarctic Slope Current (ASC). This counter current (counter to all other major Southern Ocean currents) directly feeds into the Weddell Sea (Vernet et al., 2019), Ross Sea (Roach & Speer, 2019), and Prydz Bay Gyres (Nunes Vaz & Lennon, 1996; Williams et al., 2016) and has an important impact on continental shelf water circulation, as well as heat and mass exchange at the seawater ice‐shelf exchange (Kim et al., 2016; Stark et al., 2019; Williams et al., 2016).

**Physical oceanography of the Southern Ocean and its interaction with the global ocean**

As water density increases south toward the Antarctic continent across the ACC, both temperature and salinity gradients and associated density boundaries extend across the currents, down from the surface to the seabed (Sokolov & Rintoul, 2009). These density boundaries facilitate a pathway that promotes overturning and ventilation between the surface and deep ocean interior (Morrison et al., 2015) (as much as 80% of deep water resurfaces in the Southern Ocean). North of the maximum westerly winds (~50°S), the colder surface waters subduct under the warmer surface waters and atmospheric oxygen, CO2 and heat is pumped into the global oceans (Barker & Thomas, 2004; Klinck & Nowlin, 2001). South of ~50°S, the currents, draw up nutrient rich, Circumpolar Deep Water (CDW) along these aforementioned steep density layers. This comparatively warm, upwelled Circumpolar Deep Water diverges to the north and south. The northern traveling waters are, freshened by precipitation and sea ice, warmed by the atmosphere, and eventually cross the ACC and subducts below the subtropical surface waters. The southward traveling Circumpolar Deep Water is converted to dense Antarctic Bottom Water (ABW) after it is cooled along the Antarctic coast and then sinks and flows north to fill the abyssal regions of the global oceans (Post et al., 2014; Sokolov & Rintoul, 2009; Stark et al., 2019). This system is of critical importance to the global climate system (Morrison et al., 2015) as it contributes to the meridional overturning circulation of the world's oceans (see figure 3 in Post et al., 2014).

Within the marine realm, barriers to dispersal are often less obvious and often remain poorly understood. However, the ACC has been critical in separating high Antarctic fauna from the oceans north of it (Clarke et al., 2005). The modern ACC is the world's largest ocean current and is made up of a system of fronts, currents, gyres, and overturning pathways (see Box 1) that act as the primary large‐scale dispersal vectors across the Southern Ocean (e.g., Dambach et al., 2016). Depending on the origin and direction of movement, it is unclear at what point within the water column oceanographic systems such as the ACC and Antarctic Polar Front (APF) no longer facilitate large distributions, but rather, inhibit dispersal across them (see Box 1; Figure 1). The Drake Passage and the extensive stretches of deep sea, which also separate the two continental shelves and the sub‐Antarctic islands from each other, likely also represent significant barriers to dispersal for benthic species, despite for example, traits such as eurybathy (Brey et al., 1996; Gutt, 1991).

Despite strong historical biogeographical links between benthic faunal assemblages of the Magellan region of South America and the Antarctic Peninsula (Dell, 1972), contemporary movement and gene flow in or out of the Southern Ocean and especially across the APF (Box 1; Figure 1) is extremely rare (as discussed by Clarke et al., 2005). Molecular assessments investigating genetic structure across the APF include works that revealed species‐level genetic breaks in Antarctic demersal fish (Arkhipkin et al., 2022; Shaw et al., 2004), nemertean worms (Thornhill et al., 2008), vetigastropod sea snails (González‐Wevar et al., 2021), sea spiders (Dömel et al., 2017), and even Gentoo penguins (many of which are morphologically cryptic taxa) (Vianna et al., 2017). Community‐level differences were also seen among groups of species of krill (Patarnello et al., 1996), ophiuroids (O'Hara et al., 2013) and octocorals (Dueñas et al., 2016). Excluding marine megafauna and migratory seabirds, very few taxa overcome this barrier throughout the Southern Ocean. The few exceptions include benthic marine taxa such as a sea star (Moore et al., 2018), six brittle star morpho‐species (Galaska et al., 2017a; O'Hara et al., 2013), approximately ~68 sea spider species (Dietz et al., 2019; Munilla & Membrives, 2009), an isopod species (Leese et al., 2010), and a tritoniid nudibranch (Moles et al., 2021). These examples are predominately of animals with a known dispersive larval stage. The only exceptions include the isopod, which is a brooder with long‐distance dispersal linked to rafting (Leese et al., 2010) and the direct‐developing nudibranch, *Tritonia vorax*, collected from the southern South American continental shelf and South Georgia in the Scotia Arc (Moles et al., 2021). These are remarkable findings as the prevailing APF, along with the extremes of distance, temperature, and depth, were traditionally considered to act, as an impermeable dispersal barrier for benthic organisms between the Antarctic and the more northern temperate oceans.

Life history traits are known to exert profound influence upon the connectivity of many marine organisms (e.g., Marshall et al., 2012). Overall, organisms with pelagic larval stages (planktotrophs or lecithotrophs) have greater dispersal capabilities, although the longer feeding phase of planktotrophs results in greater dispersal potential than nonfeeding lecithotrophs. Direct development is particularly common in benthic, Antarctic mollusks (Marshall et al., 2012; Moles et al., 2017; Pearse et al., 2009; Peck et al., 2006), and these larvae are protected on the benthos in their early growth; otherwise, currents such as the ACC could sweep pelagic larvae out into unfavorable environments (e.g., the deep sea) (Clarke, 1996a). Slow development is also very common for Antarctic mollusks with examples demonstrating that shelled gastropods develop thirty times slower in the Weddell Sea than their temperate relatives (Hain & Arnaud, 1993; Moran et al., 2019). These favored developmental traits may be a consequence of slow metabolism in the cold, highly stable environments in the Southern Ocean, for protection against grazing predators or to withstand the seasonal availability of light and organic matter (Moran & Woods, 2012; Peck et al., 2006). Examples among Antarctic nudibranchs include, *Bathydoris hodgsoni* (estimated development time of up to 10 years, Moles et al., 2017), and *Antarctodomus thielei* (development time of up to 8 years, Moran et al., 2019), which are two species that have the largest embryos and longest measured developmental times of any gastropods.

In this study, we investigate the benthic Southern Ocean nudibranch complex, *Doris* “*kerguelenensis*,” in which species have an estimated embryonic period of between 13 and 27 months (Hain, 1992; Moles et al., 2017; Moran et al., 2019) and produces ribbon‐like egg mass structures containing between 1500 and 2400 capsules (Moles et al., 2017). These widespread, direct‐developing, mollusks are expected to have reduced dispersal potential (Dambach et al., 2016); however, genetic studies on the *D*. “*kerguelenensis*” species complex have unveiled a significant amount of previously, undetected species‐level diversity (Maroni et al., 2022; Wilson et al., 2009, 2013). To date, 59 species within this species complex are known from Antarctica, the sub‐Antarctic Islands and the southern South American continental shelf, and are documented to occur in sympatry as well as across broad spatial scales (Maroni et al., 2022; Wilson et al., 2009, 2013). Because of the low or absent levels of recombination within mitochondrial data, due to maternal inheritance and relatively high evolutionary rate (Harrison, 1989; Moritz et al., 1987), mtDNA is a widely used tool in phylogeographic studies and can reveal information about the interconnectivity and demographic history of populations (Avise, 2000). Here, we used the protein coding mtDNA gene, cytochrome oxidase I (COI) to (i) explore the distribution patterns of six *D*. “*kerguelenensis*” species, four of which have trans‐APF distributions, one is circum‐Antarctic and one is sub‐Antarctic but also circumpolar, (ii) further examined the phylogeographic structure of three of the best‐sampled species and (iii) discuss alternative refugia use among species with the same life history traits (i.e., benthic and direct developers).

## 2 METHODS

### 2.1 Data used

To assess genetic connectivity, demography and distribution within some of the species within the *D*. “*kerguelenensis*” species complex, we analyzed previously published data from GenBank, much of it from Wilson et al. (2009) (*n* = 143; 2013, *n =* 89) and Maroni et al. (2022) (*n =* 680). Previously, species‐level entities were numbered from 1 to 59 (Maroni et al., 2022). Here, we utilize these original numbers when referring to these species‐level hypotheses. The three best‐sampled species from Maroni et al. (2022) were selected for phylogeographic insights (sp. 24, *n =* 357; sp. 28, *n =* 80; sp. 29, *n =* 280). Three additional species that were identified to span the APF (in addition to species 24) (sp. 14, *n =* 14; sp. 26, *n =* 20; sp. 42, *n =* 15) were also examined here, however due to their smaller sample sizes, no further population structure could be robustly explored. All of these specimens were collected during various Antarctic field expeditions between 2006 and 2018 from various locations in the Southern Ocean (the full collection, subsampling, extraction, and sequencing details can be found in Wilson et al., 2009, 2013, supplementary table 1 in Maroni et al., 2022; all the specimen metadata is summarized here in Appendix Table A1).

### 2.2 Haplotype structure and diversity estimates

To visually represent the geographic structure of haplotypes for each species, COI TCS (Clement et al., 2000) haplotype networks were generated in PopART (Leigh & Bryant, 2015) with a 95% probability threshold, with locality data overlaid onto each network. TCS (Clement et al., 2000) bins sequences into haplotypes, calculates the frequencies of the haplotypes within the species and estimates genealogical relationships among the haplotypes using statistical parsimony. By overlaying geographical information, we are able to visualize patterns across sampling space and overall; fifteen geographical regions were defined a priori. Due to differing depths, distances between regions, coastal currents, and ocean circulation patterns (Smith et al., 1999), we separated the Antarctic Peninsula region into four regions: (i) Palmer Archipelago, (ii) Bransfield Strait, (iii) South Shetland Islands, and (iv) Elephant Island.

For the three well‐sampled species (24, 28, and 29), levels of polymorphism in the data were represented by haplotypic (*h*) and nucleotide diversity (*π*) indices and were calculated using the R.cran (R Core Team, 2017) package “PopGenome” (Pfeifer et al., 2014) as well in DNAsp (version 6; Rozas et al., 2017). Haplotype diversity is defined as the probability that two randomly sampled alleles are different (Nei, 1987), while nucleotide diversity averages the number of nucleotide differences per site in pairwise comparisons among sequences (Nei, 1987).

### 2.3 Demographic analyses

In order to infer past population changes and/or deviations from neutrality (Rozas et al., 2017), we explored Tajima's *D* (Tajima, 1989) and Fu's *F*_s_ (Fu, 1997) statistics (10,000 permutations) using Arlequin (version 3.5; Excoffier & Lischer, 2010) (Tables 1 and 2). Tajima's *D* (*D*) and Fu's *F*_s_ (*F*_s_) were designed to distinguish between sequences evolving under neutral or non‐neutral processes (e.g. direction or balancing selection, and demographic expansion or contraction). Tajima's *D* compares the average number of pairwise differences with the number of segregating sites (Tajima, 1989) and Fu's *F*_s_ uses the distribution of alleles or haplotypes (Fu, 1997). These tests are both powerful in detecting population growth and can indicate the occurrence of population expansion.

**TABLE 1:** Genetic diversity and neutrality indices for three well‐sampled *Doris* “*kerguelenensis*” species (species 24, 28 and 29).

	Species 24	Species 28	Species 29
*n*	359	80	280
*K*	63	46	48
ss	61	50	42
p‐i	65	23	20
*h*	0.8249	0.9703	0.8594
*π*	0.00404	0.00707	0.0049
*D*	**−2.1853**	**−1.861**	−1.614
*F*_s_	0	0	0

*Note*: Significant Tajima's *D* and Fu's *F*_s_ values represented with bold. Significance *p* < .05. 10,000 permutations.

Abbreviations: *D*, Tajima's *D*; *F*_s_, Fu's *F*_s_; *h*, haplotype diversity; *K*, number of haplotypes; *N*, number of samples; p‐i, parsimony‐informative sites; ss, segregating sites; *π*, nucleotide diversity.

**TABLE 2:** Genetic diversity and neutrality indices for three well‐sampled *Doris* “*kerguelenensis*” species (species 24, 28 and 29) by sample site.

Site	Species 24						Species 28						Species 29					
	*n*	*K*	*h*	*π*	*D*	*F*_s_	*n*	*K*	*h*	*π*	*D*	*F*_s_	*n*	*K*	*h*	*π*	*D*	*F*_s_
BB	1	1	‐	‐	‐	‐	61	37	0.966	0.00542	**−2.15984**	**−38.554**						
BS	26	14	0.88	0.00277	**−1.83729**	**−11.556**							67	21	0.808	0.00509	−1.38605	**−9.011**
EL	245	30	0.717	0.00354	−1.50608	**−19.271**							51	7	0.628	0.00273	−0.72775	**−0.276**
PAL	15	6	0.762	0.00279	−0.95661	**−1.461**							104	28	0.863	0.00479	−1.09391	**−15.914**
SG	22	9	0.81	0.00327	−1.33562	**−3.329**												
SIP	1	1	‐	‐	‐	‐												
SO	12	7	0.909	0.00442	−0.22478	**−1.88**							8	5	0.857	0.00401	0.23069	**−0.731**
SR	4	2	0.5	0.00618	−0.81734	3.251												
SS	28	3	0.415	0.00078	−0.29369	**−0.221**												
SSI	4	3	0.833	0.00353	−0.78012	0.134							47	13	0.892	0.00464	−0.34773	**−2.93**
FI							1	1	‐	‐	‐	‐						
KP							18	8	0.791	0.00189	−1.46445	**−4.694**						
DS													2	2	‐	‐	‐	‐
RS													1	1	‐	‐	‐	‐
BI	1	1	‐	‐	‐	‐												

*Note*: Significant Tajima's *D* and Fu's *F*_s_ values represented with bold. Significance *p* < .05. 10,000 permutations. Location codes are as follows: BB, Burdwood Bank; BI, Bouvet Island; BS, Bransfield Strait; DS, Davis Station; EL, Elephant Island; FI, Falkland Islands; KP, Kerguelen Plateau; PAL, Palmer Archipelago; RS, Ross Sea; SG, South Georgia; SIP, Siple Island; SO, South Orkney Islands; SR, Shag Rocks; SS, South Sandwich Islands; SSI, South Shetland Islands.

Abbreviations: *D*, Tajima's *D*; *F*_s_, Fu's *F*_s_; *h*, haplotype diversity; *K*, number of haplotypes; *N*, number of samples; *π*, nucleotide diversity.

### 2.4 Intraspecific structure

Finally, Analysis of MOlecular VAriance (AMOVA) and subsequent pairwise Φ_ST_ based on 10,000 permutations were also calculated in Arlequin (version 3.5; Excoffier & Lischer, 2010) to examine genetic differentiation among sampling localities within the three well‐sampled species. When calculating the AMOVA and subsequent pairwise Φ_ST_, populations with less than three samples were omitted from the analyses.

## 3 RESULTS

### 3.1 Distributions

**FIGURE 2:**
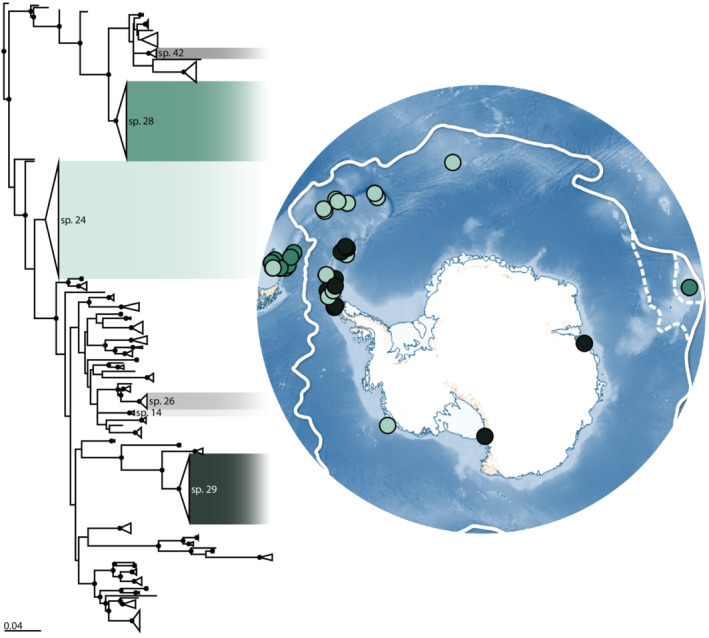
Maximum likelihood (ML) phylogeny of *Doris* “*kerguelenensis*” species 1–59 (cytochrome oxidase I gene) (left) (six boxes to highlight the species examined within this study) and map of Antarctica with sample sites of the three most well‐sampled *D*. “*kerguelenensis*” species (green shades) (right). Nodes with support values of 95 or higher have been denoted by a circular node shape. Triangles represent collapsed clades. Colored boxes indicate the six species of interest within this study (species 14, 24, 26, 28, 29, and 42). Species 14, 26, and 42 not shown on map. On the Antarctic map, the Antarctic Polar Front is denoted by the solid white line with two adjustments proposed by Park et al. (2014) and Sokolov and Rintoul (2009) (hashed white lines depicting the APF moving south of Kerguelen Island). Colors indicate species.

**FIGURE 3:**
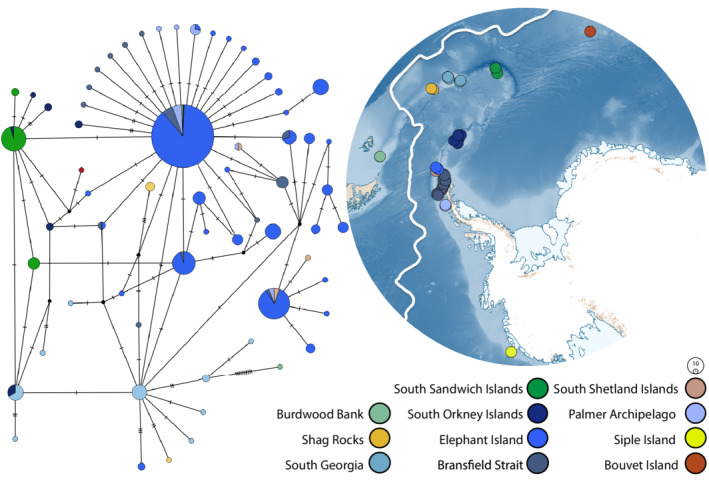
TCS haplotype network for species 24 cytochrome oxidase I data (left) (*n* = 357) and map of Antarctica (right) depicting the sample sites of species 24. The area of each circle is proportional to the frequency of the haplotype and the nodes represent unsampled or extinct haplotypes. Colors represent the location from which corresponding samples were collected. Antarctic Polar Front is denoted by solid white.

**FIGURE 4:**
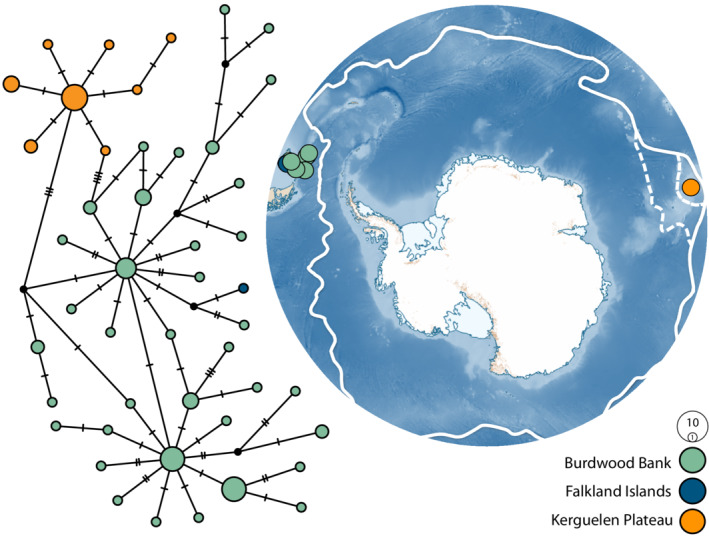
TCS haplotype network for species 28 cytochrome oxidase I data (left) (*n* = 80) and map of Antarctica (right) depicting the sample sites of species 28. The area of each circle is proportional to the frequency of the haplotype and the nodes represent unsampled or extinct haplotypes. Colors represent the location from which corresponding samples were collected. Antarctic Polar Front is denoted by solid white. With two adjustments proposed by Park et al. (2014) and Sokolov and Rintoul (2009) (hashed white lines depicting the APF moving south of Kerguelen Island).

**FIGURE 5:**
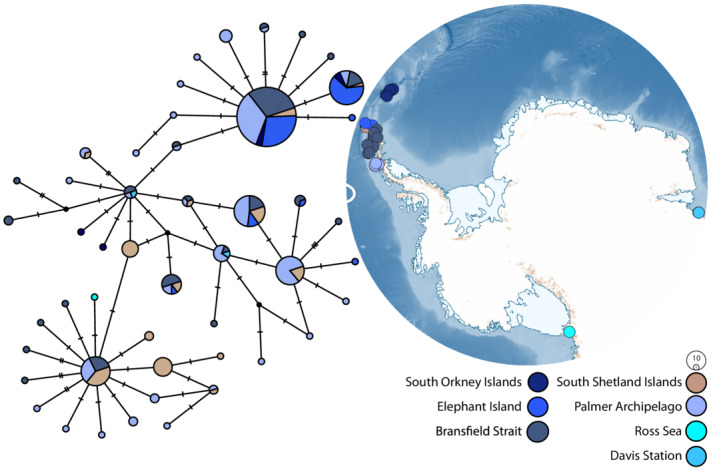
TCS haplotype network for species 29 cytochrome oxidase I data (left) (*n* = 280) and map of Antarctica (right) depicting the sample sites of species 29. The area of each circle is proportional to the frequency of the haplotype and the nodes represent unsampled or extinct haplotypes. Colors represent the location from which corresponding samples were collected.

**FIGURE 6:**
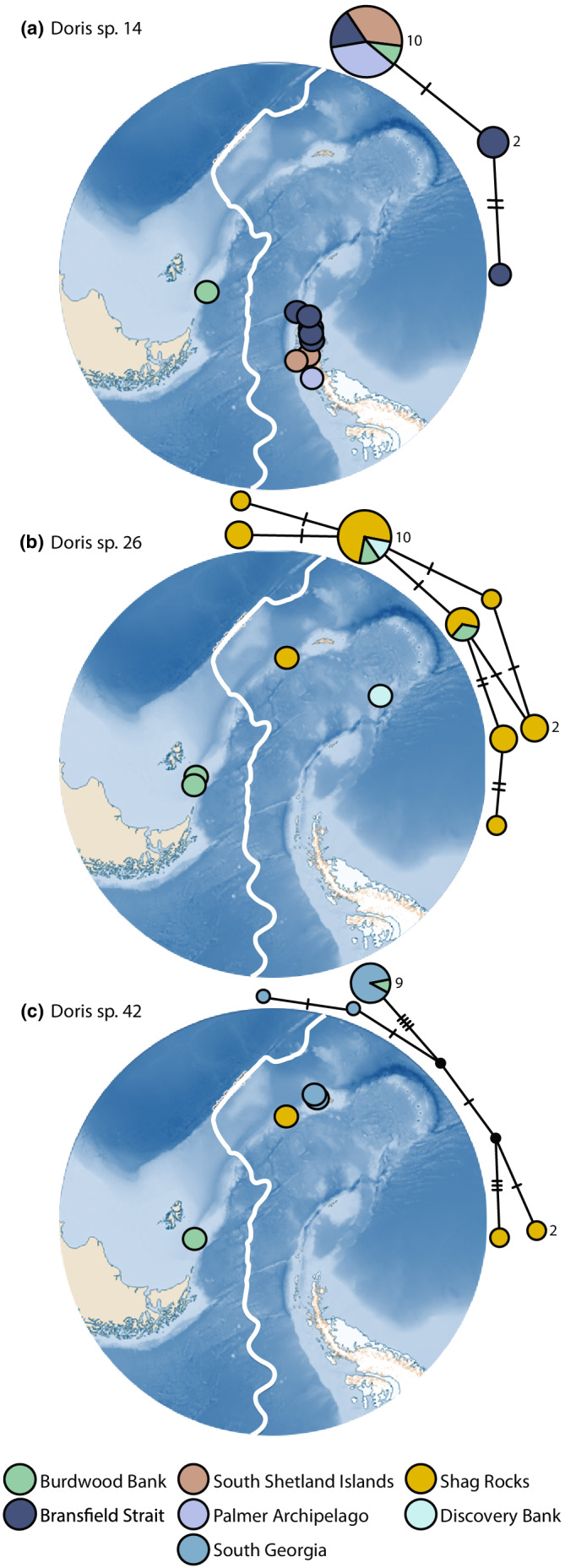
(a) TCS haplotype network for cytochrome oxidase I (COI) (right) and map of southern South America and the Antarctic Peninsula (left) depicting the sample sites of all Doris samples within species 14. The area of each circle is proportional to the frequency of the haplotype. Colors represent the location from which corresponding samples were collected. Antarctic Polar Front (APF) is denoted by solid white line. *N* = 14. (b) TCS haplotype network for COI (right) and map of southern South America and the Antarctic Peninsula (left) depicting the sample sites of all Doris samples within species 26. The area of each circle is proportional to the frequency of the haplotype. Colors represent the location from which corresponding samples were collected. APF is denoted by solid white line. *N* = 20. (c) TCS haplotype network for COI (right) and map of southern South America and the Antarctic Peninsula (left) depicting the sample sites of all Doris samples within species 42. The area of each circle is proportional to the frequency of the haplotype and the nodes represent unsampled or extinct haplotypes. Colors represent the location from which corresponding samples were collected. APF is denoted by solid white line. *N* = 15.

Within the six studied species, species 24 was the most extensively sampled (Figures 2 and 3). This species spanned the APF and was collected from over approximately 5000 km (Antarctic continental shelf, sub‐Antarctic islands, southern South American continental shelf). Species 28 (Figures 2 and 4) was only collected north of the APF, yet had a putative circum‐Antarctic distribution on the southern South American continental shelf and the Kerguelen Plateau (over a distance of approximately 7500 km). Species 29 (Figures 2 and 5) had a circum‐Antarctic distribution (collected from over 11,000 km) and was sampled from Prydz Bay, the Ross Sea, and the Antarctic Peninsula. Finally, species 14, 26, and 42 (along with sp. 24) spanned the APF near the Scotia Arc (Figure 6).

### 3.2 Haplotype and nucleotide diversity

The presence of highly represented haplotypes and many low frequency, closely related haplotypes is reflected both in the high haplotypic diversity results (species 24: *h* = 0.8249, species 28: *h* = 0.9703, species 29: *h* = 0.8594) and low nucleotide diversity (species 24: *π* = 0.00404, species 28: *π* = 0.00707, species 29: *π* = 0.0049) (Table 1). Haplotypic diversity was low within species 24 for two of the sampling locations (Shag Rocks and South Sandwich Islands). Overall, species 28 had the highest intraspecific haplotypic diversity and nucleotide diversity of the three well‐sampled species within this study. Nucleotide diversity was relatively low for each species and each location. Both indices varied within species (Table 1) and were not correlated with sample size (*p* < .05).

### 3.3 Demography

Statistical tests used for detecting demographic expansion highlighted deviations from neutrality (Tables 1 and 2). Both Tajima's *D* and Fu's *F*_s_ tests indicated significant deviations within each of the three species for at least one sampling location. Significant negative values for Tajima's *D* were determined for species 24 (Bransfield Strait) and sp. 28 (Burdwood Bank), indicating an excess or rare nucleotide site variants compared with the expectation under a neutral model of evolution, therefore suggesting population expansion (Table 2). Species 29 had a low, but not significant negative Tajima's *D* value. The results of Fu's *F*_s_ test show significant negative values for all locations for these three species (except for two poorly sampled locations within species 24). This also indicates an excess of rare haplotypes, thus supporting population expansions at most locations.

### 3.4 Intraspecific structure

When assessing genetic structure through molecular variance (Table 3) and pairwise Φ_ST_ (Tables 4, 5, 6), significant and high levels of genetic differentiation were detected within the three well‐sampled species and among all sample locations with more than three specimens (global Φ_ST_ = 0.000, *p* < .05, species 24, 28 and 29; Table 3). For species 24, genetic differentiation among sample locations represented 27.71% of the overall genetic variation, and differentiation within sample locations accounted for 72.29% of the total variance. A similar, yet exaggerated trend was seen within species 29 with only 13.95% of differentiation accounted for among locations and 83.05% within locations. For species 28, a species with no COI haplotypes shared among locations, the trend was reversed with the greater total genetic differentiation being exhibited among sample locations (58.82%), as compared to within locations (41.18%). Most pairwise Φ_ST_ tests on the populations, here defined by sampling location, within species 24 showed significant subpopulation differentiation, except for between Shag Rocks and South Shetland Islands and also among Elephant Island, the Bransfield Strait and the Palmer Archipelago (Table 4). In the case of species 28 (Table 5), significant genetic differentiation was detected between Burwood Bank and the Kerguelen Plateau (Falkland Islands omitted due to sample size). Finally, significant levels of genetic differentiation were detected between all sites within species 29 (Table 6) except between the South Orkney Islands and the Bransfield Strait, and also between the South Orkney Islands and Elephant Island.

**TABLE 3:** Analysis of molecular variance (AMOVA) between sample sites for three *Doris* “*kerguelenensis*” species (species 24, 28, and 29).

Source of variation	Degrees of freedom	Sum of squares	Variance components	Percentage of variation
Species 24				
Among populations	7	70.658	0.355 V_a_	27.71
Within populations	348	322.252	0.926 V_b_	72.29
Total	355	392.910	1.281	
Fixation indices				
Φ_ST_	0.277			
Significance tests (10,100 permutations)				
V_a_ and Φ_ST_	*p* = .000			
Species 28				
Among populations	1	59.232	2.078 V_a_	58.82
Within populations	77	112.046	1.455 V_b_	41.18
Total	78	171.278	3.534	
Fixation indices				
Φ_ST_	0.588			
Significance tests (10,100 permutations)				
V_a_ and Φ_ST_	*p* = .000			
Species 29				
Among populations	4	62.460	0.277 V_a_	13.95
Within populations	274	372.092	1.358 V_b_	83.05
Total	278	434.552	1.635	
Fixation indices				
Φ_ST_	0.169			
Significance tests (10,100 permutations)				
V_a_ and Φ_ST_	*p* = .000			

*Note*: Populations with less than three samples were removed.

**TABLE 4:** Pairwise Φ_ST_ distances (below diagonal) based on mitochondrial sequence data for 355 samples from species 24 within the *Doris* “*kerguelenensis*” species complex.

	*n*	South Shetland Islands	South Sandwich Islands	South Georgia Islands	Shag Rocks	South Orkney Island	Elephant Island	Bransfield Strait	Palmer Archipelago
South Shetland Islands	4								
South Sandwich Islands	28	**0.837**							
South Georgia	22	**0.473**	**0.55**						
Shag Rocks	4	0.569	**0.816**	**0.574**					
South Orkney Islands	12	**0.484**	**0.163**	**0.264**	**0.492**				
Elephant Island	245	**0.347**	**0.373**	**0.278**	**0.523**	**0.264**			
Bransfield Strait	26	**0.452**	**0.52**	**0.373**	**0.55**	**0.271**	0.016		
Palmer Archipelago	15	**0.437**	**0.57**	**0.358**	**0.534**	**0.256**	0.017	−0.007	

*Note*: Bold values indicate statistical significance (*p* < .05). Populations with less than three samples were removed. Populations with less than three samples were removed.

**TABLE 5:** Pairwise Φ_ST_ distances (below diagonal) based on mitochondrial sequence data for 78 samples from species 28 within the *Doris* “*kerguelenensis*” species complex.

	*n*	Burdwood Bank	Kerguelen Plateau
Burdwood Bank	61		
Kerguelen Plateau	18	**0.588**	

*Note*: Bold values indicate statistical significance (*p* < .05). Populations with less than three samples were removed. Populations with less than three samples were removed.

**TABLE 6:** Pairwise Φ_ST_ distances (below diagonal) based on mitochondrial sequence data for 278 samples from species 29 within the *Doris* “*kerguelenensis*” species complex.

	*n*	Bransfield Strait	South Shetland Islands	South Orkney Islands	Palmer Archipelago	Elephant Island
Bransfield Strait	68					
South Shetland Islands	47	**0.181**				
South Orkney Islands	8	0.008	**0.286**			
Palmer Archipelago	104	**0.015**	**0.160**	**0.082**		
Elephant Island	52	**0.148**	**0.475**	0.075	**0.210**	

*Note*: Bold values indicate statistical significance (*p* < .05). Populations with less than three samples were removed. Populations with less than three samples were removed.

### 3.5 Haplotypic network structure

Despite having a mainly parochial network reflecting geographic structure (see: Allcock & Strugnell, 2012), species 24 also shared nine haplotypes among Elephant Island, the Bransfield Strait, the Palmer Archipelago, the South Orkney Islands and Siple Island (Figure 3), which included one haplotype that was shared between all of these locations, collectively spanning ~3000 km. Of the 63 haplotypes from 357 samples, most were private (only found at a single location; *n =* 54); 32 of which were singletons. Overall, high levels of haplotypic diversity were present with a large proportion of haplotypes occurring at very low frequencies, all being only marginally differentiated from the few dominant haplotypes.

Species 28 also showed a parochial (geographically structured) network and was only collected north of the Polar Front. This species was made up of 46 haplotypes from 80 samples (Figure 4) and was collected from three locations: the Falkland Islands (*n =* 1), Burdwood Bank (*n =* 61), and the Kerguelen Plateau (*n =* 18). All haplotypes within this TCS network were private and 34 were singletons (Figure 4). Geographically proximal samples were also closely linked within the haplotype network.

Species 29 showed a diffuse network with the presence of high frequency shared haplotypes and little geographic structure. Of the 48 haplotypes determined from the 280 samples, 14 were shared among Elephant Island, the Bransfield Strait, the Palmer Archipelago, South Orkney Islands, and the South Shetland Islands (Figure 5). The remainder of the haplotypes (*n =* 34) were private, 27 of which were singletons. COI haplotypes that spanned ~9000 km were identified within this species.

Species 14 (*n =* 14), 26 (*n =* 20) and 42 (*n =* 15) were all too under sampled to infer population‐level statistics. However, these species are of interest, as all three share haplotypes across the APF (Figure 6). Together, they indicated intraspecific genetic connectivity was occurring across the APF for multiple species within the *D*. “*kerguelenensis*” species complex. Species 14 consisted of three haplotypes. One was shared between Burdwood Bank, the Bransfield Strait, the South Shetland Islands, and the Palmer Archipelago (Figure 6a). The two other haplotypes within the network were private, both occurring only in the Bransfield Strait. Species 26 consisted of eight haplotypes, two shared and six private (Figure 6b). The network of this species shows COI haplotypes spanning from Burdwood Bank to Shag Rocks and/or Discovery Bank, demonstrating some level of connectivity along the Scotia Arc. Species 42 contains four private haplotypes, plus one shared (between Burdwood Bank and South Georgia) and two unknown/under‐sampled/extinct nodes (Figure 6c). Despite being geographically close (~30 km apart), the haplotypes from South Georgia and Shag Rocks were genetically distant in the network.

## 4 DISCUSSION

### 4.1 Trans‐APF and circum‐Antarctic distributions

Rare examples of benthic, direct‐developing species spanning the APF are reported within this study. The APF is the strongest of the series of eastward‐flowing jets that make‐up the ACC; thus, the documented trans‐APF connectivity found within this study is remarkable given these animals are direct developers and lack a dispersive larval stage. Here, four species of nudibranch are documented to span this front, and three of these species are shown to share haplotypes across the front. Notably, this appears to be the first example of a benthic direct‐developing species that spans the APF from the southern South American continental shelf to the Antarctic continental shelf. Usually, the APF is considered a strong biogeographical feature that has facilitated circum‐Antarctic connectivity in some rafting taxa (e.g., Cumming et al., 2014), but also represents a pronounced barrier, which has split many evolutionary lineages between the Antarctic and lower latitudes (Clarke et al., 2005; Hunter & Halanych, 2008; Krabbe et al., 2010; Thornhill et al., 2008).

It is unclear how these direct‐developing nudibranch species have dispersed long distances, both across the APF and either through or over deep water. For animals that do not produce a free‐swimming larvae stage, this is often explained through rafting (discussed below). In our study system, we report a circum‐Antarctic species (species 29) that has a shared COI haplotype which persisted over ~9000 km (Ross Sea, Antarctic Peninsula, Davis Station). Additionally, Wilson et al. (2009) reported one shared 16S haplotype over a distance of ~6200 km. Although the presence of a single shared haplotype over long distances does not indicate direct genetic connectivity, it does appear to indicate long‐distance migration by an individual in recent history.

There are very few examples of other benthic marine taxa that are known to span the APF and of these, only two are benthic, direct‐developing taxa. One such example, *Tritonia vorax*, is a sub‐Antarctic and southern South American nudibranch (Moles et al., 2021) that was collected at shallow water depths in the Chilean fiords (13 m) and in deeper waters on the Burdwood Bank (444 m) and South Georgia, thus crossing the APF in the Scotia Sea (Moles et al., 2021; Schrödl, 1999). The Scotia Arc is the chain of islands, seamounts, and ridges that span the Scotia Sea between South America and the Antarctic Peninsula and has been implicated to act like “stepping‐stones” between the two geographic regions (Held & Agrawal, 2016). It has been hypothesized that these shallow shelf habitats have allowed dispersal across the APF, although some overlap may simply be historical rather than reflecting ongoing gene flow. Schrödl (1999) initially documented the trans‐APF distribution for *T. vorax*; however, molecular tools were not used to confirm it until recently (Moles et al., 2021). Another example that goes beyond the stepping stones of the Scotia Arc is the brooding, benthic isopod *Septemserolis septemcarinata* (Leese et al., 2010), collected from South Georgia, Bouvet Island, and Marion Island. As these isopods lack an active means of dispersal, the directional, asymmetrically‐biased (west to east) gene flow over the large geographic scales reported by Leese et al. (2010) is linked to rafting. It is also worth noting that, rather than trans‐APF distributions, some Antarctic direct developers have very large, circum‐Antarctic distributions. For example, *Nyphon australe*—a benthic, slow‐moving pycnogonid with no pelagic life stages, has a distribution encircling Antarctica (Soler‐Membrives et al., 2017), whereas for most other Antarctic sea spiders, such wide distributions have been questioned, and eventually split by the recognition of cryptic species (i.e., multiple morphologically cryptic species, rather than a single circum‐Antarctic species, e.g., Harder et al., 2016; Krabbe et al., 2010).

Across the Southern Hemisphere, there are twice as many direct‐developing species or species with nonfeeding pelagic larvae (~80%) than across the Northern Hemisphere (~30%) (Marshall et al., 2012), which may be an adaptation to particular conditions linked to Thorson's Rule (Thorson, 1936, 1946, 1950). Generally, Thorson put forward the idea that the cold Antarctic waters would be inhospitable to larvae; thus, species with pelagic larvae would be rarer in higher latitudes (Thorson, 1936, 1946, 1950). Also, larger offspring sizes are associated with lower levels of productivity and lower temperatures (Marshall et al., 2012). Long developmental times and larger offspring sizes are also well known across many other Southern Ocean taxa (Clarke, 1992, 1996b; Peck, 2016; Peck et al., 2006), and even more specifically across some Antarctic mollusks (Hain, 1992; Moles et al., 2017; Moran et al., 2019), which may be a consequence of their slow metabolisms, long glacial–interglacial periods (stable environments) and food availability (prey species are generally long‐lived). The diversity and geographic spread of *D*. “*kerguelenensis*” species clearly highlights the evolutionary success of these life history traits.

Most organisms that are documented to cross the APF are capable of rafting (Barnes, 2002; Fell, 1962; Waters, 2008), including buoyant sub‐Antarctic kelp species (e.g., *Macrocystis pyrifera* and *Durvillaea antarctica*) and kelp‐associated invertebrates (see: Cumming et al., 2014; González‐Wevar et al., 2021; Nikula et al., 2010; Waters, 2008; Waters et al., 2018). Kelp species provide suitable habitats for a diverse array of marine invertebrates and if dislodged from the benthos, will float for potentially thousands of kilometers (Fraser et al., 2009, 2017). However, there is no empirical evidence to support *D*. “*kerguelenensis*” species rafting, although passive rafting, either as egg masses on bryozoans (Wilson et al., 2009) or via anchor ice in heavily glaciated regions (Dayton et al., 1970) may provide a means for dispersal. Maroni et al. (2022) described the likelihood of *D*. “*kerguelenensis*” species rafting either as juveniles or adults on mobile benthic organisms (as well as their unlikely capacity to migrate or survive on abyssal plains), which leaves questions about their dispersal mechanisms unanswered. Leese et al. (2010) suggested that episodic long‐distance dispersal events, including one rafting subpopulation every few thousand years could ensure genetic integrity over evolutionary time scales; however, observing these migration events would be essentially impossible. Furthermore, as the migrant must be reproductively successful for gene flow to have occurred, direct measures of dispersal do not always reflect the movement of genes (Whitlock & McCauley, 1999). Our work confirms that for *D*. “*kerguelenensis*” species, long‐distance dispersal over these highly effective biogeographic barriers (the ACC, APF, geographic distance and the abyssal plains/deep‐sea) has historically been possible; however, our work does not illuminate the mechanisms or rate of such events.

### 4.2 Genetic diversity, population structure, and refugia use

Our phylogeographic analyses indicate that species 24 and 28 have a history of postglacial population expansion (significant, negative Tajima's *D* and Fu's *F*_s_ across all well‐sampled locations within these species). Due to the regions glacial history, this expansion is indicative of a bottleneck or a founder event; however, alternative hypotheses include populations expanding after a selective sweep, and/or purifying selection. As a result, the mitochondrial genetic diversity seen here may not exclusively reflect the demographic history of these tested populations and instead reflect time since a selective sweep (Bazin et al., 2006; Ellegren & Galtier, 2016; Salinas‐Ivanenko & Múrria, 2021). Within this dataset, high levels of haplotypic diversity are indicated through the generally parochial networks and associated diversity and demographic indices. Also, most haplotypes occur at low frequencies and are closely linked to the few dominant, potentially ancestral, haplotypes which is indicative of expansion. Like in other studies (Moles et al., 2021; Soler‐Membrives et al., 2017; Strugnell et al., 2012), parochial networks indicate historical geographic bottlenecks and local diversification (i.e. populations surviving and diversifying within refugia) followed by population expansion rather than after a sweep and/or purifying selection (Salinas‐Ivanenko & Múrria, 2021). This is also supported by the geographic spread of haplotypes (sites proximal to one another geographically are most similar within the network) and the lack of a higher frequency of shared haplotypes (Allcock & Strugnell, 2012). Regions with high levels of private haplotypes include the Burdwood Bank, Elephant Island, Shag Rocks, South Georgia, the South Orkney Islands, and the Kerguelen Plateau. These locations also host high levels of private haplotypes in other Antarctic taxa (see: Baird et al., 2011; González‐Wevar et al., 2021; Hemery et al., 2012; Janosik et al., 2011; Lau et al., 2021; Wilson et al., 2007, 2009) and have been proposed as glacial refugia (Lau et al., 2020).

Unlike species 24 and 28, the diffuse haplotype network structure of species 29 indicates an absence of population bottlenecks and suggests that this *Doris* species continued to disperse and diversify during glacial periods (e.g., Allcock & Strugnell, 2012). This is also supported by significant, negative Fu's *F*_s_ values. Generally, diffuse haplotype network structures have been linked with taxa that historically took refuge in the deep sea during glacial maxima. This is hard to reconcile within this study system as the present‐day distributions of *D*. “*kerguelenensis*” species do not extend beyond the continental shelf and/or slope (0–789 m; Maroni et al., 2022). Similar diffuse haplotype network structures have also been found among other animals that historically, were also not found in the deep sea (González‐Wevar et al., 2011; Hunter & Halanych, 2008; Janosik et al., 2011; Matschiner et al., 2009).

Overall, this work demonstrates that Antarctic genetic diversity and structure cannot necessarily be predicted, even between closely related species with the presumed same life history traits and dispersal capabilities (e.g., Marko, 2004; Wilson et al., 2007). With an increase in sampling (total specimen number as well as across appropriate spatial scales), species‐level histories are beginning to be realized. For example, the Antarctic brittle star *Ophinotus victoriae*, previously characterized as multiple cryptic species (from limited and disjunct sampling) is now known to represent a single widespread species (Galaska et al., 2017b; Hunter & Halanych, 2010; Lau et al., 2021). Also, regional sampling of the Antarctic crinoid *Promachocrinus kerguelensis* indicated species were not widespread (Wilson et al., 2007) but a large increase in spatial coverage showed that all seven species in the complex were circum‐Antarctic (Hemery et al., 2012). The comprehensive sample sizes of some of the *Doris* species investigated throughout this study, especially with respect to geographic scale, has improved our understanding by elucidating some of the phylogeographic signatures of this complex of direct developers.

## 5 CONCLUSIONS

*Doris* “*kerguelenensis*” is a key group of species for understanding the strong climatic events and environmental conditions that have shaped the Antarctic benthos across time. The genetic structure of *D*. “*kerguelenensis*” species' provided evidence to show intraspecific genetic connectivity occurred across the APF for multiple species within this complex. Here, we also presented evidence for a benthic, direct developer that has a present‐day distribution spanning both Antarctic and South American continental shelves. Importantly, the increase in sampling revealed extremely large distributions (exceeding thousands of kilometers) in some species. Through this sampling increase, we have highlighted how difficult it is to make generalizations about Antarctic species, even among closely related species. Here, genetic signatures between sympatric sister‐species with the same life history traits (i.e., benthic and direct developers) presented alternate phylogeographic histories that demonstrated that an increase in sampling may provide unexpected insights into many previously examined, yet undersampled species. As this work draws its conclusions from a single mitochondrial locus, we suggest that future studies instead sequence larger portions of the genome in order to produce more robust inferences about population‐level divergence, gene flow, persistence and change over time. Gaining insights into contemporary phylogeographic structure across the Antarctic region is important, since the mechanisms that have historically facilitated the evolution of such high levels of Antarctic benthic diversity may be lost in the future.

## AUTHOR CONTRIBUTIONS

**Paige J. Maroni:** Data curation (equal); formal analysis (lead); funding acquisition (equal); investigation (equal); resources (supporting); visualization (lead); writing – original draft (lead); writing – review and editing (equal). **Nerida G. Wilson:** Conceptualization (lead); data curation (equal); funding acquisition (equal); resources (lead); writing – review and editing (equal).

## FUNDING INFORMATION

The work was supported by the Society of Australian Systematic Biologists (SASB), the University of Western Australia Oceans Institute (UWA‐OI) Robson and Robertson award, the Malacological Society of Australasia (MSA), and the Antarctic Science Foundation (ASF). This work was also supported by ARC SRIEAS Grant SR200100005 Securing Antarctica's Environmental Future. These funding bodies did not have a role in the design of the study or the collection, analysis, and interpretation of data, or in writing the manuscript.

## CONFLICT OF INTEREST

We have no conflicts of interest to declare.

## Data Availability

The datasets analyzed during the current study are available from the corresponding author on reasonable request. All specimen and sequence data for this article can be found in Appendix Table A1. COI sequences are available through GenBank (https://www.ncbi.nlm.nih.gov/) ON419127–ON419135. Dryad accession number https://doi.org/10.5061/dryad.kwh70rz6w.

## Appendix

**TABLE A1:** All specimen metadata for the six individual *Doris* “*kerguelenensis*” species included in this study.

Sample ID	Species	Collection locality	Location code	Latitude	Longitude	Depth (m)	Year collected	Field/cruise code	Genbank COI
SIOBICM17430	14	King George Island	SSI	−61°20′20.4″	−55°37′30″	192	2012	PS79	ON178854
SIOBICM17471	14	King George Island	SSI	−61°20′20.4″	−55°37′30″	192	2012	PS79	ON178855
WAMS103604	14	Low Island	SSI	−63°16′59.999″	−62°9′0″		2010	LMG‐SC10	ON178856
WAMS103985	14	Bransfield Strait	BS	−62°49′0.3″	−56°39′28.62″	35–76	2006	AMLR 2006	ON178857
WAMS103669	14	Bonaparte Point	PAL	−64°46′40.681″	−64°4′1.981″	38	2018	PSC_2018	ON178858
G233_3_14	14	Bransfield Strait	BS	−63°40′8.699″	−61°10′2.82″	126	2006	LMG06‐05	EU823195
USNM1121299	14	Bransfield Strait	BS	−62°22′8.519″	−55°37′9.181″	258	2006	AMLR 2006 ‐ Leg II	EU823160
ZSM20012286‐1	14	Bransfield Strait	BS	−63°4′41.999″	−57°31′36.001″	95	2000	EASIZ 3	EU823160
WAMS103548	14	Litchfield Island	PAL	−64°45′40.619″	−64°5′27.359″		2010	PSC10‐10	ON178859
WAMS103547	14	Litchfield Island	PAL	−64°45′40.619″	−64°5′27.359″		2010	PSC10‐10	ON178860
WAMS103936	14	Janus Island	PAL	−64°47′6.421″	−64°6′7.499″		2010	PSC10‐10	ON178861
SIOBICM17562	14	Bransfield Strait	BS	−62°52′10.2″	−57°13′0.48″	247–150	2011	NBP11‐05	ON178862
SIOBICM17585	14	Burdwood Bank	BB	−54°39′23.76″	−60°1′36.48″	195–199	2011	NBP11‐05	ON178863
WAMS104004	14	Low Island	SSI	−63°16′59.999″	−62°9′0″		2010	LMG‐SC10‐130	ON178864
SIOBICM12156	24	Coronation Island	SOI	−60°57′7.2″	−45°19′44.4″	236	2009	AMLR 2009	ON178930
SIOBICM12195	24	Coronation Island	SOI	−60°45′7.2″	−44°11′56.4″	166	2009	AMLR 2009	ON178931
SIOBICM12209	24	Coronation Island	SOI	−60°42′43.2″	−46°1′1.2″	96	2009	AMLR 2009	ON178932
YPMIZ047467	24	Coronation Island	SOI	−60°29′40.452″	−44°37′51.744″	798	2009	AMLR 2009	ON178933
YPMIZ047430	24	Coronation Island	SOI	−60°35′43.08″	−44°45′39.24″	118	2009	AMLR 2009	ON419127
YPMIZ047427	24	Coronation Island	SOI	−60°35′43.08″	−44°45′39.24″	118	2009	AMLR 2009	ON178934
SIOBICM17539	24	South Georgia	SG	−55°2′20.4″	−35°26′52.8″	124–125	2011	NBP11‐05	ON178935
SIOBICM17540	24	South Georgia	SG	−55°3′3.6″	−35°23′42″	119	2011	NBP11‐05	ON178936
SIOBICM17541	24	South Georgia	SG	−55°3′3.6″	−35°23′42″	119	2011	NBP11‐05	ON178937
SIOBICM17542	24	South Georgia	SG	−53°48′25.2″	−37°13′1.2″	140–144	2011	NBP11‐05	ON178938
SIOBICM17543	24	South Georgia	SG	−53°48′25.2″	−37°13′1.2″	140–144	2011	NBP11‐05	ON178939
SIOBICM17550	24	South Georgia	SG	−53°48′0″	−37°13′8.4″	143–145	2011	NBP11‐05	ON178940
SIOBICM17547	24	South Georgia	SG	−53°48′0″	−37°13′8.4″	143–145	2011	NBP11‐05	ON178941
SIOBICM17134	24	Elephant Island	EI	−61°9′32.4″	−56°1′19.2″	150	2012	PS79	ON419132
SIOBICM17135	24	Elephant Island	EI	−61°9′32.4″	−56°1′19.2″	150	2012	PS79	ON178942
SIOBICM17147	24	Elephant Island	EI	−61°9′32.4″	−56°1′19.2″	150	2012	PS79	ON178943
SIOBICM17148	24	Elephant Island	EI	−61°9′32.4″	−56°1′19.2″	150	2012	PS79	ON178944
SIOBICM17149	24	Elephant Island	EI	−61°9′32.4″	−56°1′19.2″	150	2012	PS79	ON178945
SIOBICM17151	24	Elephant Island	EI	−61°9′32.4″	−56°1′19.2″	150	2012	PS79	ON178946
SIOBICM17152	24	Elephant Island	EI	−61°9′32.4″	−56°1′19.2″	150	2012	PS79	ON178947
SIOBICM17153	24	Elephant Island	EI	−61°9′32.4″	−56°1′19.2″	150	2012	PS79	ON178948
SIOBICM17154	24	Elephant Island	EI	−61°9′32.4″	−56°1′19.2″	150	2012	PS79	ON178949
SIOBICM17155	24	Elephant Island	EI	−61°9′32.4″	−56°1′19.2″	150	2012	PS79	ON178950
SIOBICM17156	24	Elephant Island	EI	−61°9′32.4″	−56°1′19.2″	150	2012	PS79	ON178951
SIOBICM17157	24	Elephant Island	EI	−61°9′32.4″	−56°1′19.2″	150	2012	PS79	ON419133
SIOBICM17158	24	Elephant Island	EI	−61°9′32.4″	−56°1′19.2″	150	2012	PS79	ON178952
SIOBICM17160	24	Elephant Island	EI	−61°9′32.4″	−56°1′19.2″	150	2012	PS79	ON178953
SIOBICM17161	24	Elephant Island	EI	−61°9′32.4″	−56°1′19.2″	150	2012	PS79	ON178954
SIOBICM17166	24	Elephant Island	EI	−61°9′32.4″	−56°1′19.2″	150	2012	PS79	ON178955
SIOBICM17167	24	Elephant Island	EI	−61°9′32.4″	−56°1′19.2″	150	2012	PS79	ON178956
SIOBICM17168	24	Elephant Island	EI	−61°9′32.4″	−56°1′19.2″	150	2012	PS79	ON178957
SIOBICM17169	24	Elephant Island	EI	−61°9′32.4″	−56°1′19.2″	150	2012	PS79	ON178958
SIOBICM17170	24	Elephant Island	EI	−61°9′32.4″	−56°1′19.2″	150	2012	PS79	ON178959
SIOBICM17171	24	Elephant Island	EI	−61°9′32.4″	−56°1′19.2″	150	2012	PS79	ON178960
SIOBICM17172	24	Elephant Island	EI	−61°9′32.4″	−56°1′19.2″	150	2012	PS79	ON178961
SIOBICM17173	24	Elephant Island	EI	−61°9′32.4″	−56°1′19.2″	150	2012	PS79	ON178962
SIOBICM17174	24	Elephant Island	EI	−61°9′32.4″	−56°1′19.2″	150	2012	PS79	ON178963
SIOBICM17183	24	Elephant Island	EI	−61°9′32.4″	−56°1′19.2″	150	2012	PS79	ON178964
SIOBICM17184	24	Elephant Island	EI	−61°9′32.4″	−56°1′19.2″	150	2012	PS79	ON178965
SIOBICM17185	24	Elephant Island	EI	−61°9′32.4″	−56°1′19.2″	150	2012	PS79	ON178966
SIOBICM17186	24	Elephant Island	EI	−61°9′32.4″	−56°1′19.2″	150	2012	PS79	ON178967
SIOBICM17187	24	Elephant Island	EI	−61°9′32.4″	−56°1′19.2″	150	2012	PS79	ON178968
SIOBICM17188	24	Elephant Island	EI	−61°9′32.4″	−56°1′19.2″	150	2012	PS79	ON178969
SIOBICM17189	24	Elephant Island	EI	−61°9′32.4″	−56°1′19.2″	150	2012	PS79	ON178970
SIOBICM17191	24	Elephant Island	EI	−61°9′32.4″	−56°1′19.2″	150	2012	PS79	ON178971
SIOBICM17192	24	Elephant Island	EI	−61°9′32.4″	−56°1′19.2″	150	2012	PS79	ON178972
SIOBICM17193	24	Elephant Island	EI	−61°9′32.4″	−56°1′19.2″	150	2012	PS79	ON178973
SIOBICM17194	24	Elephant Island	EI	−61°9′32.4″	−56°1′19.2″	150	2012	PS79	ON178974
SIOBICM17195	24	Elephant Island	EI	−61°9′32.4″	−56°1′19.2″	150	2012	PS79	ON178975
SIOBICM17197	24	Elephant Island	EI	−61°9′32.4″	−56°1′19.2″	150	2012	PS79	ON419131
SIOBICM17198	24	Elephant Island	EI	−61°9′32.4″	−56°1′19.2″	150	2012	PS79	ON178976
SIOBICM17199	24	Elephant Island	EI	−61°9′32.4″	−56°1′19.2″	150	2012	PS79	ON178977
SIOBICM17200	24	Elephant Island	EI	−61°9′32.4″	−56°1′19.2″	150	2012	PS79	ON178978
SIOBICM17201	24	Elephant Island	EI	−61°9′32.4″	−56°1′19.2″	150	2012	PS79	ON178979
SIOBICM17202	24	Elephant Island	EI	−61°9′32.4″	−56°1′19.2″	150	2012	PS79	ON178980
SIOBICM17203	24	Elephant Island	EI	−61°9′32.4″	−56°1′19.2″	150	2012	PS79	ON178981
SIOBICM17204	24	Elephant Island	EI	−61°9′32.4″	−56°1′19.2″	150	2012	PS79	ON178982
SIOBICM17205	24	Elephant Island	EI	−61°9′32.4″	−56°1′19.2″	150	2012	PS79	ON178983
SIOBICM17207	24	Elephant Island	EI	−61°9′32.4″	−56°1′19.2″	150	2012	PS79	ON178984
SIOBICM17208	24	Elephant Island	EI	−61°9′32.4″	−56°1′19.2″	150	2012	PS79	ON178985
SIOBICM17209	24	Elephant Island	EI	−61°9′32.4″	−56°1′19.2″	150	2012	PS79	ON178986
SIOBICM17210	24	Elephant Island	EI	−61°9′32.4″	−56°1′19.2″	150	2012	PS79	ON178987
SIOBICM17211	24	Elephant Island	EI	−61°9′32.4″	−56°1′19.2″	150	2012	PS79	ON178988
SIOBICM17212	24	Elephant Island	EI	−61°9′32.4″	−56°1′19.2″	150	2012	PS79	ON178989
SIOBICM17213	24	Elephant Island	EI	−61°9′32.4″	−56°1′19.2″	150	2012	PS79	ON178990
SIOBICM17214	24	Elephant Island	EI	−61°9′32.4″	−56°1′19.2″	150	2012	PS79	ON178991
SIOBICM17215	24	Elephant Island	EI	−61°9′32.4″	−56°1′19.2″	150	2012	PS79	ON178992
SIOBICM17216	24	Elephant Island	EI	−61°9′32.4″	−56°1′19.2″	150	2012	PS79	ON178993
SIOBICM17217	24	Elephant Island	EI	−61°9′32.4″	−56°1′19.2″	150	2012	PS79	ON178994
SIOBICM17218	24	Elephant Island	EI	−61°9′32.4″	−56°1′19.2″	150	2012	PS79	ON178995
SIOBICM17220	24	Elephant Island	EI	−61°9′32.4″	−56°1′19.2″	150	2012	PS79	ON178996
SIOBICM17221	24	Elephant Island	EI	−61°9′32.4″	−56°1′19.2″	150	2012	PS79	ON178997
SIOBICM17222	24	Elephant Island	EI	−61°9′32.4″	−56°1′19.2″	150	2012	PS79	ON178998
SIOBICM17223	24	Elephant Island	EI	−61°9′32.4″	−56°1′19.2″	150	2012	PS79	ON178999
SIOBICM17224	24	Elephant Island	EI	−61°9′32.4″	−56°1′19.2″	150	2012	PS79	ON179000
SIOBICM17225	24	Elephant Island	EI	−61°9′32.4″	−56°1′19.2″	150	2012	PS79	ON179001
SIOBICM17226	24	Elephant Island	EI	−61°9′32.4″	−56°1′19.2″	150	2012	PS79	ON179002
SIOBICM17227	24	Elephant Island	EI	−61°9′32.4″	−56°1′19.2″	150	2012	PS79	ON179003
SIOBICM17228	24	Elephant Island	EI	−61°9′32.4″	−56°1′19.2″	150	2012	PS79	ON179004
SIOBICM17229	24	Elephant Island	EI	−61°9′32.4″	−56°1′19.2″	150	2012	PS79	ON179005
SIOBICM17230	24	Elephant Island	EI	−61°9′32.4″	−56°1′19.2″	150	2012	PS79	ON179006
SIOBICM17231	24	Elephant Island	EI	−61°9′32.4″	−56°1′19.2″	150	2012	PS79	ON179007
SIOBICM17232	24	Elephant Island	EI	−61°9′32.4″	−56°1′19.2″	150	2012	PS79	ON179008
SIOBICM17233	24	Elephant Island	EI	−61°9′32.4″	−56°1′19.2″	150	2012	PS79	ON179009
SIOBICM17234	24	Elephant Island	EI	−61°9′32.4″	−56°1′19.2″	150	2012	PS79	ON179010
SIOBICM17235	24	Elephant Island	EI	−61°9′32.4″	−56°1′19.2″	150	2012	PS79	ON179011
SIOBICM17236	24	Elephant Island	EI	−61°9′32.4″	−56°1′19.2″	150	2012	PS79	ON179012
SIOBICM17237	24	Elephant Island	EI	−61°9′32.4″	−56°1′19.2″	150	2012	PS79	ON179013
SIOBICM17238	24	Elephant Island	EI	−61°9′32.4″	−56°1′19.2″	150	2012	PS79	ON179014
SIOBICM17239	24	Elephant Island	EI	−61°9′32.4″	−56°1′19.2″	150	2012	PS79	ON179015
SIOBICM17243	24	Elephant Island	EI	−61°9′32.4″	−56°1′19.2″	150	2012	PS79	ON179016
SIOBICM17244	24	Elephant Island	EI	−61°9′32.4″	−56°1′19.2″	150	2012	PS79	ON179017
SIOBICM17245	24	Elephant Island	EI	−61°9′32.4″	−56°1′19.2″	150	2012	PS79	ON179018
SIOBICM17250	24	Elephant Island	EI	−61°9′32.4″	−56°1′19.2″	150	2012	PS79	ON179019
SIOBICM17251	24	Elephant Island	EI	−61°9′32.4″	−56°1′19.2″	150	2012	PS79	ON179020
SIOBICM17252	24	Elephant Island	EI	−61°9′32.4″	−56°1′19.2″	150	2012	PS79	ON179021
SIOBICM17253	24	Elephant Island	EI	−61°9′32.4″	−56°1′19.2″	150	2012	PS79	ON179022
SIOBICM17258	24	Elephant Island	EI	−61°9′32.4″	−56°1′19.2″	150	2012	PS79	ON179023
SIOBICM17259	24	Elephant Island	EI	−61°9′32.4″	−56°1′19.2″	150	2012	PS79	ON179024
SIOBICM17260	24	Elephant Island	EI	−61°9′32.4″	−56°1′19.2″	150	2012	PS79	ON179025
SIOBICM17261	24	Elephant Island	EI	−61°9′32.4″	−56°1′19.2″	150	2012	PS79	ON179026
SIOBICM17266	24	Elephant Island	EI	−61°9′32.4″	−56°1′19.2″	150	2012	PS79	ON179027
SIOBICM17267	24	Elephant Island	EI	−61°9′32.4″	−56°1′19.2″	150	2012	PS79	ON179028
SIOBICM17268	24	Elephant Island	EI	−61°9′32.4″	−56°1′19.2″	150	2012	PS79	ON179029
SIOBICM17274	24	Elephant Island	EI	−61°9′32.4″	−56°1′19.2″	150	2012	PS79	ON179030
SIOBICM17281	24	Elephant Island	EI	−61°2′34.8″	−55°45′28.8″	130	2012	PS79	ON179031
SIOBICM17282	24	Elephant Island	EI	−61°2′34.8″	−55°45′28.8″	130	2012	PS79	ON179032
SIOBICM17283	24	Elephant Island	EI	−61°2′34.8″	−55°45′28.8″	130	2012	PS79	ON179033
SIOBICM17288	24	Elephant Island	EI	−61°2′34.8″	−55°45′28.8″	130	2012	PS79	ON179034
SIOBICM17289	24	Elephant Island	EI	−61°2′34.8″	−55°45′28.8″	130	2012	PS79	ON179035
SIOBICM17290	24	Elephant Island	EI	−61°2′34.8″	−55°45′28.8″	130	2012	PS79	ON179036
SIOBICM17291	24	Elephant Island	EI	−61°2′34.8″	−55°45′28.8″	130	2012	PS79	ON179037
SIOBICM17312	24	Elephant Island	EI	−61°2′34.8″	−55°45′28.8″	130	2012	PS79	ON179038
SIOBICM17317	24	Elephant Island	EI	−61°2′34.8″	−55°45′28.8″	130	2012	PS79	ON179039
SIOBICM17323	24	Elephant Island	EI	−61°2′34.8″	−55°45′28.8″	130	2012	PS79	ON179040
SIOBICM17295	24	Elephant Island	EI	−61°2′34.8″	−55°45′28.8″	130	2012	PS79	ON179041
SIOBICM17324	24	Elephant Island	EI	−61°2′34.8″	−55°45′28.8″	130	2012	PS79	ON179042
SIOBICM17296	24	Elephant Island	EI	−61°2′34.8″	−55°45′28.8″	130	2012	PS79	ON179043
SIOBICM17302	24	Elephant Island	EI	−61°2′34.8″	−55°45′28.8″	130	2012	PS79	ON179044
SIOBICM17309	24	Elephant Island	EI	−61°2′34.8″	−55°45′28.8″	130	2012	PS79	ON179045
SIOBICM17311	24	Elephant Island	EI	−61°2′34.8″	−55°45′28.8″	130	2012	PS79	ON179046
SIOBICM17321	24	Elephant Island	EI	−61°2′34.8″	−55°45′28.8″	130	2012	PS79	ON179047
SIOBICM17336	24	Elephant Island	EI	−61°2′34.8″	−55°45′28.8″	130	2012	PS79	ON179048
SIOBICM17348	24	Elephant Island	EI	−61°2′34.8″	−55°45′28.8″	130	2012	PS79	ON179049
SIOBICM17334	24	Elephant Island	EI	−61°2′34.8″	−55°45′28.8″	130	2012	PS79	ON179050
SIOBICM17341	24	Elephant Island	EI	−61°2′34.8″	−55°45′28.8″	130	2012	PS79	ON179051
SIOBICM17354	24	Elephant Island	EI	−61°2′34.8″	−55°45′28.8″	130	2012	PS79	ON179052
SIOBICM17361	24	Elephant Island	EI	−61°2′34.8″	−55°45′28.8″	130	2012	PS79	ON179053
SIOBICM17386	24	Elephant Island	EI	−61°2′34.8″	−55°45′28.8″	130	2012	PS79	ON179054
SIOBICM17240	24	Elephant Island	EI	−61°9′32.4″	−56°1′19.2″	150	2012	PS79	ON179055
SIOBICM17241	24	Elephant Island	EI	−61°9′32.4″	−56°1′19.2″	150	2012	PS79	ON179056
SIOBICM17242	24	Elephant Island	EI	−61°9′32.4″	−56°1′19.2″	150	2012	PS79	ON179057
SIOBICM17246	24	Elephant Island	EI	−61°9′32.4″	−56°1′19.2″	150	2012	PS79	ON179058
SIOBICM17247	24	Elephant Island	EI	−61°9′32.4″	−56°1′19.2″	150	2012	PS79	ON179059
SIOBICM17254	24	Elephant Island	EI	−61°9′32.4″	−56°1′19.2″	150	2012	PS79	ON179060
SIOBICM17255	24	Elephant Island	EI	−61°9′32.4″	−56°1′19.2″	150	2012	PS79	ON179061
SIOBICM17256	24	Elephant Island	EI	−61°9′32.4″	−56°1′19.2″	150	2012	PS79	ON179062
SIOBICM17257	24	Elephant Island	EI	−61°9′32.4″	−56°1′19.2″	150	2012	PS79	ON179063
SIOBICM17262	24	Elephant Island	EI	−61°9′32.4″	−56°1′19.2″	150	2012	PS79	ON179064
SIOBICM17263	24	Elephant Island	EI	−61°9′32.4″	−56°1′19.2″	150	2012	PS79	ON179065
SIOBICM17264	24	Elephant Island	EI	−61°9′32.4″	−56°1′19.2″	150	2012	PS79	ON179066
SIOBICM17265	24	Elephant Island	EI	−61°9′32.4″	−56°1′19.2″	150	2012	PS79	ON179067
SIOBICM17270	24	Elephant Island	EI	−61°9′32.4″	−56°1′19.2″	150	2012	PS79	ON179068
SIOBICM17272	24	Elephant Island	EI	−61°9′32.4″	−56°1′19.2″	150	2012	PS79	ON179069
SIOBICM17273	24	Elephant Island	EI	−61°9′32.4″	−56°1′19.2″	150	2012	PS79	ON179070
SIOBICM17284	24	Elephant Island	EI	−61°9′32.4″	−56°1′19.2″	130	2012	PS79	ON179071
SIOBICM17285	24	Elephant Island	EI	−61°9′32.4″	−56°1′19.2″	130	2012	PS79	ON179072
SIOBICM17286	24	Elephant Island	EI	−61°9′32.4″	−56°1′19.2″	130	2012	PS79	ON179073
SIOBICM17287	24	Elephant Island	EI	−61°9′32.4″	−56°1′19.2″	130	2012	PS79	ON179074
SIOBICM17292	24	Elephant Island	EI	−61°9′32.4″	−56°1′19.2″	130	2012	PS79	ON179075
SIOBICM17318	24	Elephant Island	EI	−61°2′34.8″	−55°45′28.8″	130	2012	PS79	ON179076
SIOBICM17329	24	Elephant Island	EI	−61°2′34.8″	−55°45′28.8″	130	2012	PS79	ON179077
SIOBICM17342	24	Elephant Island	EI	−61°2′34.8″	−55°45′28.8″	130	2012	PS79	ON179078
SIOBICM17394	24	Elephant Island	EI	−61°2′34.8″	−55°45′28.8″	130	2012	PS79	ON179079
SIOBICM17402	24	Elephant Island	EI	−61°2′34.8″	−55°45′28.8″	130	2012	PS79	ON179080
SIOBICM17387	24	Elephant Island	EI	−61°2′34.8″	−55°45′28.8″	130	2012	PS79	ON179081
SIOBICM17395	24	Elephant Island	EI	−61°2′34.8″	−55°45′28.8″	130	2012	PS79	ON179082
SIOBICM17380	24	Elephant Island	EI	−61°2′34.8″	−55°45′28.8″	130	2012	PS79	ON179083
SIOBICM17388	24	Elephant Island	EI	−61°2′34.8″	−55°45′28.8″	130	2012	PS79	ON179084
SIOBICM17396	24	Elephant Island	EI	−61°2′34.8″	−55°45′28.8″	130	2012	PS79	ON179085
SIOBICM17404	24	Elephant Island	EI	−61°2′34.8″	−55°45′28.8″	130	2012	PS79	ON179086
SIOBICM17411	24	Elephant Island	EI	−61°2′34.8″	−55°45′28.8″	130	2012	PS79	ON179087
SIOBICM17381	24	Elephant Island	EI	−61°2′34.8″	−55°45′28.8″	130	2012	PS79	ON179088
SIOBICM17397	24	Elephant Island	EI	−61°2′34.8″	−55°45′28.8″	130	2012	PS79	ON179089
SIOBICM17405	24	Elephant Island	EI	−61°2′34.8″	−55°45′28.8″	130	2012	PS79	ON179090
SIOBICM17412	24	Elephant Island	EI	−61°2′34.8″	−55°45′28.8″	130	2012	PS79	ON179091
SIOBICM17382	24	Elephant Island	EI	−61°2′34.8″	−55°45′28.8″	130	2012	PS79	ON179092
SIOBICM17390	24	Elephant Island	EI	−61°2′34.8″	−55°45′28.8″	130	2012	PS79	ON179093
SIOBICM17398	24	Elephant Island	EI	−61°2′34.8″	−55°45′28.8″	130	2012	PS79	ON179094
SIOBICM17406	24	Elephant Island	EI	−61°2′34.8″	−55°45′28.8″	130	2012	PS79	ON179095
SIOBICM17383	24	Elephant Island	EI	−61°2′34.8″	−55°45′28.8″	130	2012	PS79	ON179096
SIOBICM17391	24	Elephant Island	EI	−61°2′34.8″	−55°45′28.8″	130	2012	PS79	ON179097
SIOBICM17399	24	Elephant Island	EI	−61°2′34.8″	−55°45′28.8″	130	2012	PS79	ON179098
SIOBICM17414	24	Elephant Island	EI	−61°2′34.8″	−55°45′28.8″	130	2012	PS79	ON179099
SIOBICM17392	24	Elephant Island	EI	−61°2′34.8″	−55°45′28.8″	130	2012	PS79	ON179100
SIOBICM17400	24	Elephant Island	EI	−61°2′34.8″	−55°45′28.8″	130	2012	PS79	ON179101
SIOBICM17415	24	Elephant Island	EI	−61°2′34.8″	−55°45′28.8″	130	2012	PS79	ON179102
SIOBICM17385	24	Elephant Island	EI	−61°2′34.8″	−55°45′28.8″	130	2012	PS79	ON179103
SIOBICM17393	24	Elephant Island	EI	−61°2′34.8″	−55°45′28.8″	130	2012	PS79	ON179104
SIOBICM17401	24	Elephant Island	EI	−61°2′34.8″	−55°45′28.8″	130	2012	PS79	ON179105
SIOBICM17470	24	King George Island	SSI	−61°20′20.4″	−55°37′30″	192	2012	PS79	ON179106
SIOBICM12157	24	Coronation Island	SOI	−60°57′7.2″	−45°19′44.4″	236	2009	AMLR 2009	ON179107
SIOBICM12176	24	Coronation Island	SOI	−60°45′57.6″	−46°17′13.2″	150	2012	PS79	ON179108
SIOBICM12190	24	Coronation Island	SOI	−60°39′10.8″	−46°17′42″	104	2012	PS79	ON179109
SIOBICM12196	24	Coronation Island	SOI	−60°45′7.2″	−44°11′56.4″	166	2012	PS79	ON179110
SIOBICM17570	24	Elephant Island	EI	−61°18′14.4″	−55°42′28.8″	170–176	2011	NBP11_05	ON179111
SIOBICM17297	24	Elephant Island	EI	−61°2′34.8″	−55°45′28.8″	130	2012	PS79	ON179112
SIOBICM17303	24	Elephant Island	EI	−61°2′34.8″	−55°45′28.8″	130	2012	PS79	ON179113
SIOBICM17315	24	Elephant Island	EI	−61°2′34.8″	−55°45′28.8″	130	2012	PS79	ON179114
SIOBICM17320	24	Elephant Island	EI	−61°2′34.8″	−55°45′28.8″	130	2012	PS79	ON179115
SIOBICM17326	24	Elephant Island	EI	−61°2′34.8″	−55°45′28.8″	130	2012	PS79	ON179116
SIOBICM17344	24	Elephant Island	EI	−61°2′34.8″	−55°45′28.8″	130	2012	PS79	ON179117
SIOBICM17349	24	Elephant Island	EI	−61°2′34.8″	−55°45′28.8″	130	2012	PS79	ON179118
SIOBICM17356	24	Elephant Island	EI	−61°2′34.8″	−55°45′28.8″	130	2012	PS79	ON179119
SIOBICM17363	24	Elephant Island	EI	−61°2′34.8″	−55°45′28.8″	130	2012	PS79	ON179120
SIOBICM17370	24	Elephant Island	EI	−61°2′34.8″	−55°45′28.8″	130	2012	PS79	ON179121
SIOBICM17376	24	Elephant Island	EI	−61°2′34.8″	−55°45′28.8″	130	2012	PS79	ON179122
SIOBICM17331	24	Elephant Island	EI	−61°2′34.8″	−55°45′28.8″	130	2012	PS79	ON179123
SIOBICM17338	24	Elephant Island	EI	−61°2′34.8″	−55°45′28.8″	130	2012	PS79	ON179124
SIOBICM17345	24	Elephant Island	EI	−61°2′34.8″	−55°45′28.8″	130	2012	PS79	ON179125
SIOBICM17377	24	Elephant Island	EI	−61°2′34.8″	−55°45′28.8″	130	2012	PS79	ON179126
SIOBICM17332	24	Elephant Island	EI	−61°2′34.8″	−55°45′28.8″	130	2012	PS79	ON179127
SIOBICM17339	24	Elephant Island	EI	−61°2′34.8″	−55°45′28.8″	130	2012	PS79	ON179128
SIOBICM17346	24	Elephant Island	EI	−61°2′34.8″	−55°45′28.8″	130	2012	PS79	ON179129
SIOBICM17351	24	Elephant Island	EI	−61°2′34.8″	−55°45′28.8″	130	2012	PS79	ON179130
SIOBICM17358	24	Elephant Island	EI	−61°2′34.8″	−55°45′28.8″	130	2012	PS79	ON179131
SIOBICM17365	24	Elephant Island	EI	−61°2′34.8″	−55°45′28.8″	130	2012	PS79	ON179132
SIOBICM17372	24	Elephant Island	EI	−61°2′34.8″	−55°45′28.8″	130	2012	PS79	ON179133
SIOBICM17333	24	Elephant Island	EI	−61°2′34.8″	−55°45′28.8″	130	2012	PS79	ON179134
SIOBICM17340	24	Elephant Island	EI	−61°2′34.8″	−55°45′28.8″	130	2012	PS79	ON179135
SIOBICM17352	24	Elephant Island	EI	−61°2′34.8″	−55°45′28.8″	130	2012	PS79	ON179136
SIOBICM17359	24	Elephant Island	EI	−61°2′34.8″	−55°45′28.8″	130	2012	PS79	ON179137
SIOBICM17366	24	Elephant Island	EI	−61°2′34.8″	−55°45′28.8″	130	2012	PS79	ON179138
SIOBICM17379	24	Elephant Island	EI	−61°2′34.8″	−55°45′28.8″	130	2012	PS79	ON179139
WAMS103606	24	Low Island	SSI	−63°16′59.999″	−62°9′0″		2010	LMG‐SC10	ON179140
SIOBICM12475	24	Bransfield Strait	BS	−63°20′34.8″	−59°54′36″	213–298	2011	NBP11_05	ON179141
WAMS103987	24	Bransfield Strait	BS	−62°49′0.3″	−56°39′28.62″		2006	AMLR 2006	USNM1120702
SIOBICM12179	24	South Orkney Islands	SOI	−61°13′37.2″	−46°23′49.2″	130	2009	AMLR 2009	ON179142
SIOBICM13058	24	Elephant Island	EI	−62°22′1.2″	−56°1′44.4″	245–266	2012	PS79	ON179143
SIOBICM13073	24	Elephant Island	EI	−62°22′1.2″	−56°1′44.4″	245–266	2012	PS79	ON179144
WAMS103726	24	Litchfield Island	PAL	−64°46′4.739″	−64°5′1.86″	15	2018	PSC_2018	ON179145
WAMS103765	24	Gamage Point	PAL	−64°46′26.699″	−64°3′23.641″	35	2018	PSC_2018	ON179146
PSC08‐06‐A	24	Janus Island	PAL	−64°47′6.421″	−64°6′7.499″	0–35	2008	PSC08‐06	JX680552
PSC08‐06‐AA	24	Hermit Island	PAL	−64°48′8.341″	−64°1′26.281″	0–35	2008	PSC08‐06	JX680551
PSC08‐06‐J	24	Janus Island	PAL	−64°47′6.421″	−64°6′7.499″	0–35	2008	PSC08‐06	JX680553
PSC08‐06‐U	24	Hermit Island	PAL	−64°48′8.341″	−64°1′26.281″	0–35	2008	PSC08‐06	JX680550
PSC08‐06‐Z	24	Hermit Island	PAL	−64°48′8.341″	−64°1′26.281″	0–35	2008	PSC08‐06	JX680554
CASIZ171176	24	Bouvet Island	BI	−54°29′24″	−3°18′0″	169	2004	ICEFISH	EU823203
USNM1121609	24	Bransfield Strait	BS	−63°13′45.001″	−58°45′20.002″	87	2004	LMG04‐14	EU823180
USNM1121613	24	Bransfield Strait	BS	−63°13′45.001″	−58°45′20.002″	87	2004	LMG04‐14	EU823138
USNM1121598	24	Bransfield Strait	BS	−63°13′45.001″	−58°45′20.002″	87	2004	LMG04‐14	EU823138
USNM1121590	24	Bransfield Strait	BS	−63°13′45.001″	−58°45′20.002″	87	2004	LMG04‐14	EU823156
USNM1121597	24	Bransfield Strait	BS	−63°13′45.001″	−58°45′20.002″	87	2004	LMG04‐14	EU823181
USNM1121621	24	Bransfield Strait	BS	−63°13′45.001″	−58°45′20.002″	87	2004	LMG04‐14	EU823183
USNM1121591	24	Bransfield Strait	BS	−63°13′45.001″	−58°45′20.002″	87	2004	LMG04‐14	EU823129
USNM1121602	24	Bransfield Strait	BS	−63°13′45.001″	−58°45′20.002″	87	2004	LMG04‐14	EU823184
USNM1120718	24	Bransfield Strait	BS	−63°23′3.001″	−60°3′24.001″	277	2004	LMG04‐14	EU823156
USNM1121611	24	Bransfield Strait	BS	−63°0′0″	−62°0′0″	192	2004	LMG04‐14	EU823196
G149_2_24	24	Bransfield Strait	BS	−63°0′0″	−62°0′0″	192	2004	LMG04‐14	EU823197
USNM1120721	24	Shag Rocks	SR	−53°45′0″	−41°28′12″	191	2004	ICEFISH	EU823134
USNM1120826	24	Shag Rocks	SR	−53°45′0″	−41°28′12″	191	2004	ICEFISH	EU823134
USNM1121605	24	Bransfield Strait	BS	−63°7′9.001″	−58°41′31.319″	150	2006	LMG06‐05	EU823156
USNM1121601	24	Bransfield Strait	BS	−63°7′9.001″	−58°41′31.319″	150	2006	LMG06‐05	EU823156
USNM1121345	24	Bransfield Strait	BS	−62°36′46.681″	−56°36′36.9″	231	2006	AMLR 2006 ‐ Leg II	EU823156
USNM1121357	24	Bransfield Strait	BS	−62°30′52.499″	−55°58′53.159″	238	2006	AMLR 2006 ‐ Leg II	EU823150
USNM1120836	24	Bransfield Strait	BS	−63°3′50.699″	−57°9′16.859″	253	2006	AMLR 2006 ‐ Leg II	EU823138
ZSM20021056	24	Elephant Island	EI	−61°2′54.6″	−55°52′46.801″	149	2002	ANDEEP 1	EU823138
WAMS103535	24	Norsel Point	PAL	−64°45′40.619″	−64°5′27.359″	27–35	2010	PSC10‐10	ON179147
WAMS103946	24	Norsel Point	PAL	−64°45′38.279″	−64°5′52.44″	27–35	2010	PSC10‐10	ON179148
WAMS103527	24	Norsel Point	PAL	−64°45′38.279″	−64°5′52.44″	27–35	2010	PSC10‐10	ON179149
WAMS103525	24	Gamage Point	PAL	−64°46′28.56″	−64°3′24.901″	20–33	2010	PSC10‐10	ON179150
WAMS103951	24	Gamage Point	PAL	−64°46′28.56″	−64°3′24.901″	20–33	2010	PSC10‐10	ON179151
SIOBICM17525	24	South Sandwich	SS	−56°43′26.4″	−27°2′9.6″	134–142	2011	NBP11_05	ON179152
SIOBICM17526	24	South Sandwich	SS	−56°43′26.4″	−27°2′9.6″	134–142	2011	NBP11_05	ON179153
SIOBICM17527	24	South Sandwich	SS	−56°43′26.4″	−27°2′9.6″	134,142	2011	NBP11_05	ON179154
SIOBICM17514	24	South Sandwich	SS	−56°42′32.4″	−27°2′56.4″	99–116	2011	NBP11_05	ON179155
SIOBICM17515	24	South Sandwich	SS	−56°42′32.4″	−27°2′56.4″	99–116	2011	NBP11_05	ON179156
SIOBICM17516	24	South Sandwich	SS	−56°42′32.4″	−27°2′56.4″	99–116	2011	NBP11_05	ON179157
SIOBICM17517	24	South Sandwich	SS	−56°42′32.4″	−27°2′56.4″	99–116	2011	NBP11_05	ON179158
SIOBICM17518	24	South Sandwich	SS	−56°42′32.4″	−27°2′56.4″	99–116	2011	NBP11_05	ON179159
SIOBICM17519	24	South Sandwich	SS	−56°42′32.4″	−27°2′56.4″	99–116	2011	NBP11_05	ON179160
SIOBICM17520	24	South Sandwich	SS	−56°42′32.4″	−27°2′56.4″	99–116	2011	NBP11_05	ON179161
SIOBICM17522	24	South Sandwich	SS	−56°42′32.4″	−27°2′56.4″	99–116	2011	NBP11_05	ON179162
SIOBICM17523	24	South Sandwich	SS	−56°42′32.4″	−27°2′56.4″	99–116	2011	NBP11_05	ON179163
SIOBICM17524	24	South Sandwich	SS	−56°42′32.4″	−27°2′56.4″	99–116	2011	NBP11_05	ON179164
SIOBICM12529	24	South Georgia	SS	−55°3′3.6″	−35°23′42″	119	2011	NBP11_05	ON179165
SIOBICM12530	24	South Georgia	SS	−55°3′3.6″	−35°23′42″	119	2011	NBP11_05	ON179166
SIOBICM12531	24	South Georgia	SS	−55°3′3.6″	−35°23′42″	119	2011	NBP11_05	ON179167
SIOBICM12526	24	South Georgia	SS	−55°2′20.4″	−35°26′52.8″	124–125	2011	NBP11_05	ON179168
SIOBICM12538	24	South Sandwich	SS	−56°42′32.4″	−27°2′56.4″	99–116	2011	NBP11_05	ON179169
SIOBICM12554	24	South Sandwich	SS	−56°43′26.4″	−27°2′9.6″	134–142	2011	NBP11_05	ON179170
SIOBICM12552	24	South Sandwich	SS	−56°43′26.4″	−27°2′9.6″	134–142	2011	NBP11_05	ON179171
SIOBICM12473	24	Bransfield Strait	BS	−63°20′34.8″	−59°54′36″	213–298	2011	NBP11_05	ON179172
SIOBICM12553	24	South Sandwich	SS	−56°43′26.4″	−27°2′9.6″	134–142	2011	NBP11_05	ON179173
SIOBICM12562	24	South Sandwich	SS	−56°43′26.4″	−27°2′9.6″	134–142	2011	NBP11_05	ON179174
SIOBICM12542	24	South Sandwich	SS	−56°42′32.4″	−27°2′56.4″	99–116	2011	NBP11_05	ON179175
SIOBICM12535	24	South Sandwich	SS	−56°42′32.4″	−27°2′56.4″	99–116	2011	NBP11_05	ON179176
SIOBICM12536	24	South Sandwich	SS	−56°42′32.4″	−27°2′56.4″	99–116	2011	NBP11_05	ON179177
SIOBICM12543	24	South Sandwich	SS	−56°42′32.4″	−27°2′56.4″	99–116	2011	NBP11_05	ON179178
SIOBICM12537	24	South Sandwich	SS	−56°42′32.4″	−27°2′56.4″	99–116	2011	NBP11_05	ON179179
SIOBICM12544	24	South Sandwich	SS	−56°42′32.4″	−27°2′56.4″	99–116	2011	NBP11_05	ON179180
SIOBICM12540	24	South Sandwich	SS	−56°42′32.4″	−27°2′56.4″	99–116	2011	NBP11_05	ON179181
SIOBICM12539	24	South Sandwich	SS	−56°42′32.4″	−27°2′56.4″	99–116	2011	NBP11_05	ON179182
SIOBICM12541	24	South Sandwich	SS	−56°42′32.4″	−27°2′56.4″	99–116	2011	NBP11_05	ON179183
SIOBICM12548	24	South Georgia	SS	−53°48′0″	−37°13′8.4″	143–145	2011	NBP11_05	ON179184
SIOBICM12547	24	South Georgia	SS	−53°48′0″	−37°13′8.4″	143–145	2011	NBP11_05	ON179185
SIOBICM12516	24	South Georgia	SS	−53°48′25.2″	−37°13′1.2″	140–144	2011	NBP11_05	ON179186
SIOBICM18250	24	Bransfield Strait	BS	−63°20′34.8″	−59°54′36″	213–298	2011	NBP11_05	ON179187
SIOBICM12556	24	Bransfield Strait	BS	−62°52′10.236″	−57°13′0.552″	247–150	2011	NBP11_05	ON179188
SIOBICM12572	24	Bransfield Strait	BS	−62°45′10.8″	−57°19′19.2″	292–272	2011	NBP11_05	ON179189
SIOBICM12500B	24	Bransfield Strait	BS	−63°19′22.8″	−59°51′3.6″	197–199	2011	NBP11_05	ON179190
SIOBICM12499B	24	Bransfield Strait	BS	−63°19′22.8″	−59°51′3.6″	197–199	2011	NBP11_05	ON179191
SIOBICM12517	24	South Georgia	SG	−53°48′25.2″	−37°13′1.2″	140–144	2011	NBP11_05	ON179192
SIOBICM12518	24	South Georgia	SG	−53°48′25.2″	−37°13′1.2″	140–144	2011	NBP11_05	ON179193
SIOBICM13198	24	South Georgia	SG	−53°38′52.8″	−37°16′26.4″	140–139	2013	NBP13_03	ON179194
SIOBICM13261	24	South Georgia	SG	−53°38′52.8″	−37°16′26.4″	140–139	2013	NBP13_03	ON179195
SIOBICM13176	24	South Georgia	SG	−53°40′37.2″	−37°14′45.6″	137	2013	NBP13_03	ON179196
SIOBICM13246	24	South Georgia	SG	−53°40′37.2″	−37°14′45.6″	137	2013	NBP13_03	ON179197
SIOBICM13206	24	Shag Rocks	SR	−53°32′6″	−41°37′12″	127–129	2013	NBP13_03	ON179198
SIOBICM13202	24	Shag Rocks	SR	−53°31′40.8″	−41°37′1.2″	125	2013	NBP13_03	ON179199
SIOBICM17410	24	Elephant Island	EI	−61°2′34.8″	−55°45′28.8″	130	2012	PS 2012	ON179200
SIOBICM17347	24	Elephant Island	EI	−61°2′34.8″	−55°45′28.8″	130	2012	PS 2012	ON179201
SIOBICM17136	24	Elephant Island	EI	−61°9′32.4″	−56°1′19.2″	150	2012	PS 2012	ON179202
SIOBICM17179	24	Elephant Island	EI	−61°9′32.4″	−56°1′19.2″	150	2012	PS 2012	ON179203
SIOBICM17129	24	South Orkney Islands	SOI	−60°37′58.8″	−46°32′16.08″	130	2009	AMLR 2009	ON179204
WAMS101096	24	Siple Island	SIP	−73°9′43.56″	−126°57′0″	300	2017	ACE 2017	ON179205
WAMS103523	24	Palmer Station	PAL	−64°47′28.1″	−64°0′52.6″	30	2010	PSC10‐10	ON179206
SIOBICM17150	24	Elephant Island	EI	−61°9′32.4″	−56°1′19.2″	150	2012	PS79	ON179207
SIOBICM17544	24	South Georgia	SG	−53°46′1.2″	−37°13′1.56″	143–151	2011	NBP11‐05	ON179208
SIOBICM17545	24	South Georgia	SG	−53°46′1.2″	−37°13′1.56″	143–151	2011	NBP11‐05	ON179209
WAMS103569	24	Low Island	SSI	−63°16′59.999″	−62°9′0″		2010	LMG‐SC10	ON179210
SIOBICM17138	24	Elephant Island	EI	−61°9′32.4″	−56°1′19.2″	150	2012	PS79	ON179211
SIOBICM17140	24	Elephant Island	EI	−61°9′32.4″	−56°1′19.2″	150	2012	PS79	ON179212
SIOBICM17145	24	Elephant Island	EI	−61°9′32.4″	−56°1′19.2″	150	2012	PS79	ON179213
SIOBICM17146	24	Elephant Island	EI	−61°9′32.4″	−56°1′19.2″	150	2012	PS79	ON179214
SIOBICM17180	24	Elephant Island	EI	−61°9′32.4″	−56°1′19.2″	150	2012	PS79	ON179215
SIOBICM17181	24	Elephant Island	EI	−61°9′32.4″	−56°1′19.2″	150	2012	PS79	ON179216
SIOBICM17182	24	Elephant Island	EI	−61°9′32.4″	−56°1′19.2″	150	2012	PS79	ON179217
SIOBICM17190	24	Elephant Island	EI	−61°9′32.4″	−56°1′19.2″	150	2012	PS79	ON179218
SIOBICM17196	24	Elephant Island	EI	−61°9′32.4″	−56°1′19.2″	150	2012	PS79	ON179219
SIOBICM17159	24	Elephant Island	EI	−61°9′32.4″	−56°1′19.2″	150	2012	PS79	ON179220
SIOBICM17162	24	Elephant Island	EI	−61°9′32.4″	−56°1′19.2″	150	2012	PS79	ON179221
SIOBICM17163	24	Elephant Island	EI	−61°9′32.4″	−56°1′19.2″	150	2012	PS79	ON179222
SIOBICM17164	24	Elephant Island	EI	−61°9′32.4″	−56°1′19.2″	150	2012	PS79	ON179223
SIOBICM17165	24	Elephant Island	EI	−61°9′32.4″	−56°1′19.2″	150	2012	PS79	ON179224
SIOBICM17176	24	Elephant Island	EI	−61°9′32.4″	−56°1′19.2″	150	2012	PS79	ON179225
SIOBICM17177	24	Elephant Island	EI	−61°9′32.4″	−56°1′19.2″	150	2012	PS79	ON179226
SIOBICM17178	24	Elephant Island	EI	−61°9′32.4″	−56°1′19.2″	150	2012	PS79	ON179227
SIOBICM17294	24	Elephant Island	EI	−61°2′34.8″	−55°45′28.8″	130	2012	PS79	ON179228
SIOBICM17299	24	Elephant Island	EI	−61°2′34.8″	−55°45′28.8″	130	2012	PS79	ON179229
SIOBICM17300	24	Elephant Island	EI	−61°2′34.8″	−55°45′28.8″	130	2012	PS79	ON179230
SIOBICM17301	24	Elephant Island	EI	−61°2′34.8″	−55°45′28.8″	130	2012	PS79	ON179231
SIOBICM17307	24	Elephant Island	EI	−61°2′34.8″	−55°45′28.8″	130	2012	PS79	ON179232
SIOBICM17308	24	Elephant Island	EI	−61°2′34.8″	−55°45′28.8″	130	2012	PS79	ON179233
SIOBICM17313	24	Elephant Island	EI	−61°2′34.8″	−55°45′28.8″	130	2012	PS79	ON179234
SIOBICM17319	24	Elephant Island	EI	−61°2′34.8″	−55°45′28.8″	130	2012	PS79	ON179235
SIOBICM17325	24	Elephant Island	EI	−61°2′34.8″	−55°45′28.8″	130	2012	PS79	ON179236
SIOBICM17304	24	Elephant Island	EI	−61°2′34.8″	−55°45′28.8″	130	2012	PS79	ON179237
SIOBICM17316	24	Elephant Island	EI	−61°2′34.8″	−55°45′28.8″	130	2012	PS79	ON179238
SIOBICM17305	24	Elephant Island	EI	−61°2′34.8″	−55°45′28.8″	130	2012	PS79	ON179239
SIOBICM17335	24	Elephant Island	EI	−61°2′34.8″	−55°45′28.8″	130	2012	PS79	ON179240
SIOBICM17355	24	Elephant Island	EI	−61°2′34.8″	−55°45′28.8″	130	2012	PS79	ON179241
SIOBICM17362	24	Elephant Island	EI	−61°2′34.8″	−55°45′28.8″	130	2012	PS79	ON179242
SIOBICM17367	24	Elephant Island	EI	−61°2′34.8″	−55°45′28.8″	130	2012	PS79	ON179243
SIOBICM17369	24	Elephant Island	EI	−61°2′34.8″	−55°45′28.8″	130	2012	PS79	ON179244
SIOBICM17374	24	Elephant Island	EI	−61°2′34.8″	−55°45′28.8″	130	2012	PS79	ON179245
SIOBICM17375	24	Elephant Island	EI	−61°2′34.8″	−55°45′28.8″	130	2012	PS79	ON179246
SIOBICM17330	24	Elephant Island	EI	−61°2′34.8″	−55°45′28.8″	130	2012	PS79	ON179247
SIOBICM17343	24	Elephant Island	EI	−61°2′34.8″	−55°45′28.8″	130	2012	PS79	ON179248
SIOBICM17353	24	Elephant Island	EI	−61°2′34.8″	−55°45′28.8″	130	2012	PS79	ON179249
SIOBICM17368	24	Elephant Island	EI	−61°2′34.8″	−55°45′28.8″	130	2012	PS79	ON179250
SIOBICM17378	24	Elephant Island	EI	−61°2′34.8″	−55°45′28.8″	130	2012	PS79	ON179251
SIOBICM17350	24	Elephant Island	EI	−61°2′34.8″	−55°45′28.8″	130	2012	PS79	ON179252
SIOBICM17595	24	South Sandwich	SS	−56°0′0″	−27°0′0″	99–116	2011	NBP11_05	ON179253
WAMS103779	24	Palmer Station	PAL	−64°46′28.689″	−64°3′16.094″		2003	PSC03	ON179254
WAMS103781	24	Palmer Station	PAL	−64°46′28.689″	−64°3′16.094″		2003	PSC03	ON179255
SIOBICM17298	24	Elephant Island	EI	−61°2′34.8″	−55°45′28.8″	130	2012	PS79	ON179256
SIOBICM18297	24	Burdwood Bank	BB	−53°53′31.2″	−60°40′40.8″	132	2013	NBP13_03	ON179257
WAMS104000	24	Low Island	SSI	−63°16′59.999″	−62°9′0″		2010	LMG‐SC10	ON179258
SIOBICM17534	26	Shag Rocks	SR	−53°32′2.4″	−41°38′2.4″	132–133	2011	NBP11‐05	ON419130
SIOBICM13269	26	Shag Rocks	SR	−53°32′34.8″	−41°37′33.6″	130–127	2013	NBP13_03	ON179280
SIOBICM17530	26	Shag Rocks	SR	−53°31′44.4″	−41°38′2.4″	128–132	2011	NBP11_05	ON179281
SIOBICM12522	26	Shag Rocks	SR	−53°34′30″	−41°40′44.4″	134–136	2011	NBP11_05	ON179282
SIOBICM12533	26	Shag Rocks	SR	−53°31′44.4″	−41°38′2.4″	128–132	2011	NBP11_05	ON179283
SIOBICM12434	26	Shag Rocks	SR	−53°31′44.4″	−41°38′2.4″	587–610	2011	NBP11_05	ON179284
SIOBICM12524	26	Shag Rocks	SR	−53°32′2.4″	−41°38′2.4″	132–133	2011	NBP11_05	ON179285
SIOBICM13216	26	Shag Rocks	SR	−53°32′56.4″	−41°39′3.6″	127–129	2013	NBP13_03	ON179286
SIOBICM13250	26	Shag Rocks	SR	−53°32′56.4″	−41°39′3.6″	127–129	2013	NBP13_03	ON179287
SIOBICM13196	26	Shag Rocks	SR	−53°33′32.4″	−41°39′14.4″	130–128	2013	NBP13_03	ON179288
SIOBICM13189	26	Shag Rocks	SR	−53°32′56.4″	−41°38′6″	127–128	2013	NBP13_03	ON179289
SIOBICM13255	26	Shag Rocks	SR	−53°32′56.4″	−41°38′6″	127–128	2013	NBP13_03	ON179290
SIOBICM13259	26	Shag Rocks	SR	−53°31′40.8″	−41°37′1.2″	125	2013	NBP13_03	ON179291
SIOBICM13243	26	Shag Rocks	SR	−53°31′40.8″	−41°37′1.2″	125	2013	NBP13_03	ON179292
SIOBICM13181	26	Shag Rocks	SR	−53°31′58.8″	−41°37′4.8″	126–128	2013	NBP13_03	ON179293
SIOBICM13283	26	Burdwood Bank	BB	−53°53′34.8″	−60°43′12″	131–134	2013	NBP13_03	ON179294
SIOBICM17557	26	Discovery Bank	DB	−60°7′38.64″	−34°54′11.16″	379–392	2011	NBP11‐05	ON179295
SIOBICM13299	26	Burdwood Bank	BB	−53°58′0.12″	−61°27′55.8″	159	2013	Austrodoris NBP13_03	ON179296
SIOBICM13291	26	Shag Rocks	SR	−53°33′1.8″	−41°39′7.92″	128–129	2013	Austrodoris NBP13_03	ON179297
SIOBICM13294	26	Shag Rocks	SR	−53°32′56.04″	−41°38′4.2″	127–128	2013	Austrodoris NBP13_03	ON179298
SIOBICM13182	28	Burdwood Bank	BB	−54°33′32.4″	−56°49′44.4″	87–93	2013	NBP13_03	ON179301
SIOBICM13298	28	Falkland Island	FI	−52°28′48″	−60°24′54″	153–155	2013	NBP13_03	ON179302
CASIZ171180b	28	Burdwood Bank	BB	−54°31′12″	−56°37′12″	125	2004	ICEFISH	EU823129
USNM1121608	28	Burdwood Bank	BB	−54°49′0.001″	−60°16′0.001″	110	2006	LMG06‐05	EU823127
USNM1120707	28	Burdwood Bank	BB	−54°41′25.001″	−59°23′30.998″	207	2006	LMG04‐14	EU823128
ZSM20021059‐1	28	Burdwood Bank	BB	−54°31′13.199″	−56°8′55.799″	287	2002	LAMPOS	EU823205
ZSM20021059‐3	28	Burdwood Bank	BB	−54°31′13.199″	−56°8′55.799″	287	2002	LAMPOS	EU823206
ZSM20021249	28	Burdwood Bank	BB	−54°30′13.201″	−56°8′12.001″	289	2002	LAMPOS	EU823217
SIOBICM12566	28	Burdwood Bank	BB	−54°39′25.2″	−60°1′37.2″	195–199	2011	NBP11_05	ON179303
SIOBICM12508	28	Burdwood Bank	BB	−54°40′19.2″	−60°57′43.2″	176–183	2011	NBP11_05	ON179304
SIOBICM13207	28	Burdwood Bank	BB	−54°33′36″	−56°49′48″	90–92	2013	NBP13_03	ON179305
SIOBICM13214	28	Burdwood Bank	BB	−54°33′57.6″	−56°53′45.6″	96–97	2013	NBP13_03	ON179306
SIOBICM13265	28	Burdwood Bank	BB	−54°33′57.6″	−56°53′45.6″	96–97	2013	NBP13_03	ON179307
SIOBICM13210	28	Burdwood Bank	BB	−54°33′57.6″	−56°53′45.6″	96–97	2013	NBP13_03	ON179308
SIOBICM13284	28	Burdwood Bank	BB	−54°33′57.6″	−56°53′45.6″	96–97	2013	NBP13_03	ON179309
SIOBICM13205	28	Burdwood Bank	BB	−54°32′38.4″	−56°42′50.4″	108	2013	NBP13_03	ON179310
SIOBICM18263	28	Burdwood Bank	BB	54°32′52.8″	−56°45′43.2″	94–102	2013	NBP13_03	ON179311
SIOBICM13183	28	Burdwood Bank	BB	−54°33′57.6″	−56°53′45.6″	96–97	2013	NBP13_03	ON179312
SIOBICM13282	28	Burdwood Bank	BB	−54°32′56.4″	−56°44′6″	108–109	2013	NBP13_03	ON179313
SIOBICM18273	28	Burdwood Bank	BB	−53°54′3.6″	−61°28′22.8″	190–193	2013	NBP13_03	ON179314
SIOBICM18276	28	Burdwood Bank	BB	−53°54′3.6″	−61°28′22.8″	190–193	2013	NBP13_03	ON179315
SIOBICM18277	28	Burdwood Bank	BB	−53°54′3.6″	−61°28′22.8″	190–193	2013	NBP13_03	ON179316
SIOBICM18278	28	Burdwood Bank	BB	−53°54′3.6″	−61°28′22.8″	190–193	2013	NBP13_03	ON179317
SIOBICM18280	28	Burdwood Bank	BB	−53°54′0″	−61°28′22.8″	189–195	2013	NBP13_03	ON179318
SIOBICM18281	28	Burdwood Bank	BB	−53°54′0″	−61°28′22.8″	189–195	2013	NBP13_03	ON179319
SIOBICM13278	28	Burdwood Bank	BB	−53°54′0″	−61°28′22.8″	189–195	2013	NBP13_03	ON179320
SIOBICM18282	28	Burdwood Bank	BB	−53°53′42″	−61°28′15.6″	188–193	2013	NBP13_03	ON179321
SIOBICM13237	28	Burdwood Bank	BB	−53°54′3.6″	−61°28′22.8″	190–193	2013	NBP13_03	ON179322
SIOBICM13236	28	Burdwood Bank	BB	−53°54′3.6″	−61°28′22.8″	190–193	2013	NBP13_03	ON179323
SIOBICM13267	28	Burdwood Bank	BB	−53°53′52.8″	−61°28′26.4″	192–195	2013	NBP13_03	ON179324
SIOBICM18283	28	Burdwood Bank	BB	−53°53′42″	−61°28′15.6″	188–193	2013	NBP13_03	ON179325
SIOBICM18284	28	Burdwood Bank	BB	−53°53′42″	−61°28′15.6″	188–193	2013	NBP13_03	ON179326
SIOBICM13217	28	Burdwood Bank	BB	−53°53′42″	−61°28′15.6″	192–195	2013	NBP13_03	ON179327
SIOBICM18285	28	Burdwood Bank	BB	−53°53′52.8″	−61°28′26.4″	192–195	2013	NBP13_03	ON179328
SIOBICM18286	28	Burdwood Bank	BB	−53°53′52.8″	−61°28′26.4″	192–195	2013	NBP13_03	ON179329
SIOBICM13232	28	Burdwood Bank	BB	−53°53′52.8″	−61°28′26.4″	192–195	2013	NBP13_03	ON179330
SIOBICM13274	28	Burdwood Bank	BB	−53°53′52.8″	−61°28′26.4″	192–195	2013	NBP13_03	ON179331
SIOBICM13253	28	Burdwood Bank	BB	−53°58′1.2″	−61°27′54″	159	2013	NBP13_03	ON179332
SIOBICM13251	28	Burdwood Bank	BB	−53°58′1.2″	−61°27′54″	159	2013	NBP13_03	ON179333
SIOBICM18287	28	Burdwood Bank	BB	−53°58′8.4″	−61°28′1.2″	158–160	2013	NBP13_03	ON179334
SIOBICM13280	28	Burdwood Bank	BB	−53°58′8.4″	−61°28′1.2″	158–160	2013	NBP13_03	ON179335
SIOBICM13258	28	Burdwood Bank	BB	−53°58′1.2″	−61°27′54″	159	2013	NBP13_03	ON179336
SIOBICM18289	28	Burdwood Bank	BB	−53°54′36″	−61°27′25.2″	180–181	2013	NBP13_03	ON179337
SIOBICM13270	28	Burdwood Bank	BB	−53°54′36″	−61°27′25.2″	180–181	2013	NBP13_03	ON419128
SIOBICM13289	28	Burdwood Bank	BB	−53°54′36″	−61°27′25.2″	180–181	2013	NBP13_03	ON179338
SIOBICM13287	28	Burdwood Bank	BB	−53°54′36″	−61°27′25.2″	180–181	2013	NBP13_03	ON179339
SIOBICM18291	28	Burdwood Bank	BB	−53°58′8.4″	−61°28′8.4″	158–160	2013	NBP13_03	ON179340
SIOBICM13249	28	Burdwood Bank	BB	−53°58′8.4″	−61°28′8.4″	160–161	2013	NBP13_03	ON179341
SIOBICM13220	28	Burdwood Bank	BB	−53°58′8.4″	−61°28′8.4″	157–158	2013	NBP13_03	ON179342
SIOBICM13241	28	Burdwood Bank	BB	−53°58′8.4″	−61°28′8.4″	157–158	2013	NBP13_03	ON179343
SIOBICM18292	28	Burdwood Bank	BB	−53°58′8.4″	−61°28′8.4″	157–158	2013	NBP13_03	ON179344
SIOBICM13186	28	Burdwood Bank	BB	−53°58′8.4″	−61°28′8.4″	157–158	2013	NBP13_03	ON179345
SIOBICM13227	28	Burdwood Bank	BB	−53°57′50.4″	−61°28′12″	161–162	2013	NBP13_03	ON179346
SIOBICM13262	28	Burdwood Bank	BB	−53°57′50.4″	−61°28′12″	161–162	2013	NBP13_03	ON179347
SIOBICM13286	28	Burdwood Bank	BB	−53°57′50.4″	−61°28′12″	161–162	2013	NBP13_03	ON179348
SIOBICM18298	28	Burdwood Bank	BB	−53°53′31.2″	−60°40′51.6″	153–155	2013	NBP13_03	ON179349
SIOBICM13279	28	Burdwood Bank	BB	−53°54′7.2″	−61°26′2.4″	176–180	2013	NBP13_03	ON179350
SIOBICM13226	28	Burdwood Bank	BB	−53°54′7.2″	−61°26′2.4″	176–180	2013	NBP13_03	ON179351
SIOBICM13295	28	Burdwood Bank	BB	−54°33′57.6″	−56°53′44.16″	96–97	2013	Austrodoris NBP13_03	ON179352
SIOBICM13180	28	Burdwood Bank	BB	−54°32′56.4″	−56°44′6″	108–109	2013	NBP13_03	ON179353
SIOBICM18290	28	Burdwood Bank	BB	−53°54′36″	−61°27′25.2″	180–181	2013	NBP13_03	ON179354
SIOBICM13242	28	Burdwood Bank	BB	−53°58′8.4″	−61°28′8.4″	160–161	2013	NBP13_03	ON179355
WAMS71752	28	Kerguelen Plateau	KP	−49°21′9″	70°13′4.98″	4	2016	Proteker 5/PTK5_06	ON179356
WAMS71754	28	Kerguelen Plateau	KP	−49°21′9″	70°13′4.98″	4	2016	Proteker 5/PTK5_06	ON179357
WAMS71755	28	Kerguelen Plateau	KP	−49°21′9″	70°13′4.98″	4	2016	Proteker 5/PTK5_06	ON179358
WAMS71756	28	Kerguelen Plateau	KP	−49°21′9″	70°13′4.98″	4	2016	Proteker 5/PTK5_06	ON179359
WAMS71757	28	Kerguelen Plateau	KP	−49°21′9″	70°13′4.98″	4	2016	Proteker 5/PTK5_06	ON179360
WAMS71758	28	Kerguelen Plateau	KP	−49°21′9″	70°13′4.98″	4	2016	Proteker 5/PTK5_06	ON179361
WAMS71759	28	Kerguelen Plateau	KP	−49°21′9″	70°13′4.98″	4	2016	Proteker 5/PTK5_06	ON179362
WAMS71760	28	Kerguelen Plateau	KP	−49°21′9″	70°13′4.98″	4	2016	Proteker 5/PTK5_06	ON179363
WAMS71761	28	Kerguelen Plateau	KP	−49°21′9″	70°13′4.98″	4	2016	Proteker 5/PTK5_06	ON179364
WAMS71762	28	Kerguelen Plateau	KP	−49°21′9″	70°13′4.98″	4	2016	Proteker 5/PTK5_06	ON179365
WAMS71763	28	Kerguelen Plateau	KP	−49°21′9″	70°13′4.98″	4	2016	Proteker 5/PTK5_06	ON179366
WAMS71764	28	Kerguelen Plateau	KP	−49°22′57.36″	70°11′8.52″	4	2016	Proteker 5/PTK5_14	ON179367
WAMS71765	28	Kerguelen Plateau	KP	−49°22′57.36″	70°11′8.52″	4	2016	Proteker 5/PTK5_14	ON179368
WAMS71766	28	Kerguelen Plateau	KP	−49°22′57.36″	70°11′8.52″	4	2016	Proteker 5/PTK5_14	ON179369
WAMS71767	28	Kerguelen Plateau	KP	−49°22′57.36″	70°11′8.52″	4	2016	Proteker 5/PTK5_14	ON179370
WAMS71769	28	Kerguelen Plateau	KP	−49°22′57.36″	70°11′8.52″	4	2016	Proteker 5/PTK5_14	ON179371
WAMS71753	28	Kerguelen Plateau	KP	−49°21′9″	70°13′4.98″	4	2016	Proteker 5/PTK5_06	ON179372
WAMS71768	28	Kerguelen Plateau	KP	−49°21′9″	70°13′4.98″	4	2016	Proteker 5/PTK5_06	ON179373
WAMS103504	29	Palmer Station	PAL	−64°46′28.499″	−64°3′17.341″	18	2010	PSC10‐10	ON179374
WAMS103511	29	Lemaire Island	PAL	−65°4′37.081″	−63°58′10.859″		2010	PSC10‐10	ON179375
WAMS103516	29	Lemaire Island	PAL	−65°5′58.2″	−63°59′14.701″		2010	PSC10‐10	ON179376
WAMS103522	29	Christie Cove	PAL	−64°47′28.14″	−64°0′52.621″	30	2010	PSC10‐10	ON179377
WAMS103524	29	Christie Cove	PAL	−64°47′28.14″	−64°0′52.621″	30	2010	PSC10‐10	ON179378
WAMS103531	29	Palmer Station	PAL	−64°46′57.601″	−64°2′59.161″	18–33	2010	PSC10‐10	ON179379
WAMS103546	29	Spume Island	PAL	−64°47′55.741″	−64°6′45.479″		2010	PSC10‐10	ON179380
WAMS103553	29	Janus Island	PAL	−64°46′57.601″	−64°2′59.161″		2010	PSC10‐10	ON179381
WAMS103554	29	Shortcut Island	PAL	−64°46′57.601″	−64°2′59.161″		2010	PSC10‐10	ON179382
SIOBICM17576	29	Bransfield Strait	BS	−62°51′54″	−57°12′39.6″	161–163	2011	NBP11‐05	ON179383
SIOBICM17578	29	Bransfield Strait	BS	−62°51′54″	−57°12′39.6″	161–163	2011	NBP11‐05	ON179384
SIOBICM17175	29	Elephant Island	EI	−61°9′32.4″	−56°1′19.2″	150	2012	PS79	ON179385
SIOBICM17206	29	Elephant Island	EI	−61°9′32.4″	−56°1′19.2″	150	2012	PS79	ON179386
SIOBICM17219	29	Elephant Island	EI	−61°9′32.4″	−56°1′19.2″	150	2012	PS79	ON179387
SIOBICM17269	29	Elephant Island	EI	−61°9′32.4″	−56°1′19.2″	150	2012	PS79	ON179388
SIOBICM17248	29	Elephant Island	EI	−61°9′32.4″	−56°1′19.2″	150	2012	PS79	ON179389
SIOBICM17249	29	Elephant Island	EI	−61°9′32.4″	−56°1′19.2″	150	2012	PS79	ON179390
SIOBICM17271	29	Elephant Island	EI	−61°9′32.4″	−56°1′19.2″	150	2012	PS79	ON179391
SIOBICM17409	29	Elephant Island	EI	−61°2′34.8″	−55°45′28.8″	130	2012	PS79	ON179392
SIOBICM17389	29	Elephant Island	EI	−61°2′34.8″	−55°45′28.8″	130	2012	PS79	ON179393
SIOBICM17384	29	Elephant Island	EI	−61°2′34.8″	−55°45′28.8″	130	2012	PS79	ON179394
SIOBICM17408	29	Elephant Island	EI	−61°2′34.8″	−55°45′28.8″	130	2012	PS79	ON179395
SIOBICM17463	29	King George Island	SSI	−61°20′20.4″	−55°37′30″	192	2012	PS79	ON179396
SIOBICM17450	29	King George Island	SSI	−61°20′20.4″	−55°37′30″	192	2012	PS79	ON179397
SIOBICM17451	29	King George Island	SSI	−61°20′20.4″	−55°37′30″	192	2012	PS79	ON179398
SIOBICM17452	29	King George Island	SSI	−61°20′20.4″	−55°37′30″	192	2012	PS79	ON179399
SIOBICM17453	29	King George Island	SSI	−61°20′20.4″	−55°37′30″	192	2012	PS79	ON179400
SIOBICM17454	29	King George Island	SSI	−61°20′20.4″	−55°37′30″	192	2012	PS79	ON179401
SIOBICM17455	29	King George Island	SSI	−61°20′20.4″	−55°37′30″	192	2012	PS79	ON179402
SIOBICM17456	29	King George Island	SSI	−61°20′20.4″	−55°37′30″	192	2012	PS79	ON179403
SIOBICM17458	29	King George Island	SSI	−61°20′20.4″	−55°37′30″	192	2012	PS79	ON179404
SIOBICM17442	29	King George Island	SSI	−61°20′20.4″	−55°37′30″	192	2012	PS79	ON179405
SIOBICM17443	29	King George Island	SSI	−61°20′20.4″	−55°37′30″	192	2012	PS79	ON179406
SIOBICM17444	29	King George Island	SSI	−61°20′20.4″	−55°37′30″	192	2012	PS79	ON179407
SIOBICM17445	29	King George Island	SSI	−61°20′20.4″	−55°37′30″	192	2012	PS79	ON179408
SIOBICM17446	29	King George Island	SSI	−61°20′20.4″	−55°37′30″	192	2012	PS79	ON179409
SIOBICM17449	29	King George Island	SSI	−61°20′20.4″	−55°37′30″	192	2012	PS79	ON179410
SIOBICM17432	29	King George Island	SSI	−61°20′20.4″	−55°37′30″	192	2012	PS79	ON179411
SIOBICM17433	29	King George Island	SSI	−61°20′20.4″	−55°37′30″	192	2012	PS79	ON179412
SIOBICM17434	29	King George Island	SSI	−61°20′20.4″	−55°37′30″	192	2012	PS79	ON179413
SIOBICM17435	29	King George Island	SSI	−61°20′20.4″	−55°37′30″	192	2012	PS79	ON179414
SIOBICM17436	29	King George Island	SSI	−61°20′20.4″	−55°37′30″	192	2012	PS79	ON179415
SIOBICM17438	29	King George Island	SSI	−61°20′20.4″	−55°37′30″	192	2012	PS79	ON179416
SIOBICM17439	29	King George Island	SSI	−61°20′20.4″	−55°37′30″	192	2012	PS79	ON179417
SIOBICM17440	29	King George Island	SSI	−61°20′20.4″	−55°37′30″	192	2012	PS79	ON179418
SIOBICM17424	29	King George Island	SSI	−61°20′20.4″	−55°37′30″	192	2012	PS79	ON179419
SIOBICM17426	29	King George Island	SSI	−61°20′20.4″	−55°37′30″	192	2012	PS79	ON179420
SIOBICM17427	29	King George Island	SSI	−61°20′20.4″	−55°37′30″	192	2012	PS79	ON179421
SIOBICM17428	29	King George Island	SSI	−61°20′20.4″	−55°37′30″	192	2012	PS79	ON179422
SIOBICM17429	29	King George Island	SSI	−61°20′20.4″	−55°37′30″	192	2012	PS79	ON179423
SIOBICM17431	29	King George Island	SSI	−61°20′20.4″	−55°37′30″	192	2012	PS79	ON179424
SIOBICM17468	29	King George Island	SSI	−61°20′20.4″	−55°37′30″	192	2012	PS79	ON179425
SIOBICM17469	29	King George Island	SSI	−61°20′20.4″	−55°37′30″	192	2012	PS79	ON179426
SIOBICM17460	29	King George Island	SSI	−61°20′20.4″	−55°37′30″	192	2012	PS79	ON179427
WAMS103574	29	Palmer Station	PAL	−64°46′28.499″	−64°3′17.341″	18	2010	PSC10‐10	ON179428
WAMS103575	29	Lemaire Island	PAL	−65°4′37.081″	−63°58′10.859″	18	2010	PSC10‐10	ON179429
WAMS103587	29	Spume Island	PAL	−64°47′55.741″	−64°6′45.479″	30	2010	PSC10‐10	ON179430
WAMS103588	29	Spume Island	PAL	−64°47′55.741″	−64°6′45.479″		2010	PSC10‐10	ON179431
WAMS103589	29	Spume Island	PAL	−64°47′28.14″	−64°0′52.621″		2010	PSC10‐10	ON179432
SIOBICM12175	29	Coronation Island	SOI	−60°45′57.6″	−46°17′13.2″	150	2012	PS79	ON179433
SIOBICM12191	29	Coronation Island	SOI	−60°39′10.8″	−46°17′42″	104	2012	PS79	ON179434
SIOBICM12197	29	Coronation Island	SOI	−60°45′7.2″	−44°11′56.4″	166	2012	PS79	ON179435
SIOBICM12203	29	Coronation Island	SOI	−60°45′7.2″	−44°11′56.4″	166	2012	PS79	ON179436
YPMIZ047429	29	South Orkney Islands	SOI	−60°35′43.08″	−44°45′39.24″	118	2009	AMLR 2009	ON179437
SIOBICM17563	29	Bransfield Strait	BS	−62°52′10.236″	−57°13′0.552″	150–247	2011	NBP11_05	ON179438
SIOBICM17567	29	Bransfield Strait	BS	−62°52′10.236″	−57°13′0.552″	150–247	2011	NBP11_05	ON179439
SIOBICM17582	29	Bransfield Strait	BS	−62°52′10.236″	−57°13′0.552″	150–247	2011	NBP11_05	ON179440
SIOBICM17310	29	Elephant Island	EI	−61°2′34.8″	−55°45′28.8″	130	2012	PS79	ON179441
SIOBICM17357	29	Elephant Island	EI	−61°2′34.8″	−55°45′28.8″	130	2012	PS79	ON179442
WAMS103994	29	Unknown	UK				2013	NBP13_03	ON179443
SIOBICM17373	29	Elephant Island	EI	−61°2′34.8″	−55°45′28.8″	130	212	PS79	ON179444
WAMS103593	29	Laggard Island	PAL	−64°48′22.021″	−64°0′56.459″	30	2010	PSC10‐10	ON179445
WAMS103595	29	Litchfield Island	PAL	−64°45′40.619″	−64°5′27.359″		2010	PSC10‐10	ON179446
WAMS103599	29	Low Island	SSI	−63°16′59.999″	−62°9′0″		2010	LMG‐SC10	ON179447
WAMS103602	29	Low Island	SSI	−63°16′59.999″	−62°9′0″		2010	LMG‐SC10	ON179448
WAMS103609	29	Low Island	SSI	−63°16′59.999″	−62°9′0″		2010	LMG‐SC10	ON179449
WAMS103610	29	Low Island	SSI	−63°16′59.999″	−62°9′0″		2010	LMG‐SC10	ON179450
WAMS103620	29	Plow Islands	DS	−68°31′43.32″	78°3′38.52″	0–35	2010	Australian Antarctic Division	ON179451
WAMS103649	29	Livingston Island	SSI	−62°37′12.601″	−60°21′20.999″	Dec‐18	2019	BLUEBIO‐2	ON179452
WAMS103909	29	Bonaparte Island	PAL	−64°46′39.72″	−64°3′59.159″		2010	PSC10‐10	ON179453
WAMS103907	29	Norsel Point	PAL	−64°45′38.279″	−64°5′52.44″		2010	PSC10‐10	ON179454
WAMS103657	29	Beaumount Skerries	PAL	−64°46′28.139″	−64°18′33.3″	35	2018	PSC_2018	ON179455
WAMS103724	29	Casey (Nightmare) Islands	PAL	−64°43′40.98″	−64°14′54.841″	29	2018	PSC_2018	ON179456
WAMS103700	29	De Laca Island	PAL	−64°46′45.599″	−64°5′56.699″	27	2018	PSC_2018	ON179457
WAMS103738	29	De Laca Island	PAL	−64°46′45.599″	−64°5′56.699″	27	2018	PSC_2018	ON179458
WAMS103721	29	De Laca Island	PAL	−64°46′45.599″	−64°5′56.699″	27	2018	PSC_2018	ON179459
WAMS103795	29	Bahia Paraiso	PAL	−64°46′49.62″	−64°5′44.999″		2017	PSC_2017	ON179460
WAMS103845	29	Bonaparte Point	PAL	−64°46′44.821″	−64°46′44.821″		2017	PSC_2017	ON179461
WAMS103652	29	Palmer Station	PAL	−64°45′41.879″	−64°5′29.159″	39	2018	PSC_2018	ON179462
WAMS103662	29	Laggard Island	PAL	−64°48′21.539″	−64°0′56.819″	36	2018	PSC_2018	ON179463
WAMS103663	29	Palmer Station	PAL	−64°46′26.699″	−64°3′23.641″		2018	PSC_2018	ON179464
WAMS103664	29	Bonaparte Point	PAL	−64°46′40.681″	−64°4′1.981″	38	2018	PSC_2018	ON179465
WAMS103665	29	Allan's Wall	PAL	−64°45′41.879″	−64°5′29.159″	39	2018	PSC_2018	ON179466
WAMS103681	29	Bonaparte Point	PAL	−64°46′40.681″	−64°4′1.981″	38	2018	PSC_2018	ON179467
WAMS103703	29	Gamage Point	PAL	−64°46′26.699″	−64°3′23.641″	35	2018	PSC_2018	ON179468
WAMS103722	29	Stepping Stones	PAL	−64°47′10.561″	−63°59′49.621″	33	2018	PSC_2018	ON179469
WAMS103727	29	Gamage Point	PAL	−64°46′26.699″	−64°3′23.641″	35	2018	PSC_2018	ON179470
WAMS103730	29	Gamage Point	PAL	−64°46′26.699″	−64°3′23.641″	35	2018	PSC_2018	ON179471
WAMS103733	29	Palmer Dock	PAL	−64°46′28.441″	−64°3′16.801″	24	2018	PSC_2018	ON179472
WAMS103735	29	Janus Island	PAL	−64°47′5.1″	−64°5′56.699″	38	2018	PSC_2018	ON179473
WAMS103758	29	Gamage Point	PAL	−64°46′26.699″	−64°3′23.641″	35	2018	PSC_2018	ON179474
WAMS103766	29	Bonaparte Point	PAL	−64°46′40.681″	−64°4′1.981″	6	2018	PSC_2018	ON179475
WAMS103767	29	Bonaparte Point	PAL	−64°46′40.681″	−64°4′1.981″	6	2018	PSC_2018	ON179476
WAMS103773	29	Gamage Point	PAL	−64°46′26.699″	−64°3′23.641″	35	2018	PSC_2018	ON179477
WAMS103777	29	Gamage Point	PAL	−64°46′26.699″	−64°3′23.641″	35	2018	PSC_2018	ON179478
WAMS103782	29	Palmer Station	PAL	−64°46′28.689″	−64°3′16.094″		2003	PSC03	ON179479
WAMS103784	29	Palmer Station	PAL	−64°46′28.689″	−64°3′16.094″		2003	PSC03	ON179480
WAMS103791	29	Hermit Island	PAL	−64°47′55.021″	−64°0′26.341″		2017	PSC_2017	ON179481
WAMS103792	29	Norsel Point	PAL	−64°45′40.68″	−64°5′28.68″		2017	PSC_2017	ON179482
WAMS103794	29	Laggard Island	PAL	−64°48′22.14″	−64°0′56.639″	35	2017	PSC_2017	ON179483
WAMS103797	29	Gamage Point	PAL	−64°46′26.641″	−64°3′23.94″		2017	PSC_2017	ON179484
WAMS103798	29	Allan's Wall	PAL	−64°45′40.68″	−64°5′28.68″		2017	PSC_2017	ON179485
WAMS103800	29	Litchfield Island	PAL	−64°45′59.339″	−64°6′1.141″		2017	PSC_2017	ON179486
WAMS103801	29	Laggard Island	PAL	−64°48′22.14″	−64°0′56.639″	35	2017	PSC_2017	ON179487
WAMS103804	29	Litchfield Island	PAL	−64°45′59.339″	−64°6′1.141″		2017	PSC_2017	ON179488
WAMS103805	29	Allan's Wall	PAL	−64°45′40.68″	−64°5′28.68″		2017	PSC_2017	ON179489
WAMS103806	29	Allan's Wall	PAL	−64°45′40.68″	−64°5′28.68″		2017	PSC_2017	ON179490
WAMS103807	29	Gamage Point	PAL	−64°46′26.641″	−64°3′23.94″		2017	PSC_2017	ON179491
WAMS103808	29	Gamage Point	PAL	−64°46′26.641″	−64°3′23.94″		2017	PSC_2017	ON179492
WAMS103812	29	Gamage Point	PAL	−64°46′26.641″	−64°3′23.94″		2017	PSC_2017	ON179493
WAMS103813	29	Litchfield Island	PAL	−64°46′4.739″	−64°5′1.86″		2017	PSC_2017	ON179494
WAMS103817	29	Litchfield Island	PAL	−64°45′59.339″	−64°6′1.141″		2017	PSC_2017	ON179495
WAMS103821	29	Bonaparte Point	PAL	−64°46′44.821″	−64°46′44.821″		2017	PSC_2017	ON179496
WAMS103825	29	Litchfield Island	PAL	−64°46′3.659″	−64°5′36.719″		2017	PSC_2017	ON179497
WAMS103826	29	Laggard Island	PAL	−64°48′22.14″	−64°0′56.639″	35	2017	PSC_2017	ON179498
WAMS103829	29	Laggard Island	PAL	−64°48′22.14″	−64°0′56.639″	35	2017	PSC_2017	ON179499
WAMS103830	29	Bonaparte Point	PAL	−64°46′44.821″	−64°46′44.821″		2017	PSC_2017	ON179500
WAMS103834	29	Litchfield Island	PAL	−64°46′4.739″	−64°5′1.86″		2017	PSC_2017	ON179501
WAMS103835	29	Gamage Point	PAL	−64°46′26.641″	−64°3′23.94″		2017	PSC_2017	ON179502
WAMS103836	29	Litchfield Island	PAL	−64°45′59.339″	−64°6′1.141″		2017	PSC_2017	ON179503
WAMS103837	29	Litchfield Island	PAL	−64°45′59.339″	−64°6′1.141″		2017	PSC_2017	ON179504
WAMS103841	29	Litchfield Island	PAL	−64°45′59.339″	−64°6′1.141″		2017	PSC_2017	ON179505
WAMS103851	29	Laggard Island	PAL	−64°48′22.14″	−64°0′56.639″	35	2017	PSC_2017	ON179506
WAMS103852	29	Laggard Island	PAL	−64°48′22.14″	−64°0′56.639″	35	2017	PSC_2017	ON179507
WAMS103857	29	Litchfield Island	PAL	−64°46′3.659″	−64°5′36.719″		2017	PSC_2017	ON179508
WAMS103859	29	Allan's Wall	PAL	−64°45′40.68″	−64°5′28.68″		2017	PSC_2017	ON179509
WAMS103860	29	Stepping Stones Island	PAL	−64°47′1.619″	−63°59′27.841″	31	2017	PSC_2017	ON179510
WAMS103867	29	Stepping Stones Island	PAL	−64°47′1.619″	−63°59′27.841″		2017	PSC_2017	ON179511
WAMS103868	29	Laggard Island	PAL	−64°48′22.14″	−64°0′56.639″	35	2017	PSC_2017	ON179512
WAMS103871	29	Janus Island	PAL	−64°47′4.92″	−64°5′58.859″		2017	PSC_2017	ON179513
WAMS103888	29	Laggard Island	PAL	−64°48′22.14″	−64°0′56.639″		2017	PSC_2017	ON179514
WAMS103891	29	Gamage Point	PAL	−64°46′26.641″	−64°3′23.94″		2017	PSC_2017	ON179515
WAMS103892	29	Litchfield Island	PAL	−64°45′59.339″	−64°6′1.141″		2017	PSC_2017	ON179516
WAMS103894	29	Bonaparte Point	PAL	−64°46′44.821″	−64°46′44.821″		2017	PSC_2017	ON179517
WAMS103897	29	Bonaparte Point	PAL	−64°46′44.821″	−64°46′44.821″		2017	PSC_2017	ON179518
WAMS103898	29	Gamage Point	PAL	−64°46′26.641″	−64°3′23.94″		2017	PSC_2017	ON179519
WAMS103666	29	Bonaparte Point	PAL	−64°46′40.681″	−64°4′1.981″	38	2018	PSC_2018	ON179520
WAMS103725	29	Gamage Point	PAL	−64°46′26.699″	−64°3′23.641″	35	2018	PSC_2018	ON179521
WAMS103769	29	Gamage Point	PAL	−64°46′26.699″	−64°3′23.641″	35	2017	PSC_2017	ON179522
WAMS103793	29	Allan's Wall	PAL	−64°45′40.68″	−64°5′28.68″		2017	PSC_2017	ON179523
WAMS103895	29	Allan's Wall	PAL	−64°45′40.68″	−64°5′28.68″		2017	PSC_2017	ON179524
PSC08‐06‐AC	29	Hermit Island	PAL	−64°48′8.341″	−64°1′26.281″	0–35	2008	PSC08‐06	JX680556
PSC08‐06‐AF	29	Hermit Island	PAL	−64°48′8.341″	−64°1′26.281″	0–35	2008	PSC08‐06	JX680557
PSC08‐06‐AK	29	Laggard Island	PAL	−64°48′34.441″	−64°0′59.159″	0–35	2008	PSC08‐06	JX680558
PSC08‐06‐AN	29	Bonaparte Point	PAL	−64°46′45.239″	−64°2′39.419″	0–35	2008	PSC08‐06	JX680559
PSC08‐06‐AT	29	Hero Inlet	PAL	−64°46′28.38″	−64°3′17.039″	0–35	2008	PSC08‐06	JX680560
PSC08‐06‐AU	29	Hero Inlet	PAL	−64°46′28.38″	−64°3′17.039″	0–35	2008	PSC08‐06	JX680561
PSC08‐06‐AV	29	Hero Inlet	PAL	−64°46′28.38″	−64°3′17.039″	0–35	2008	PSC08‐06	JX680562
PSC08‐06‐AW	29	Hero Inlet	PAL	−64°46′28.38″	−64°3′17.039″	0–35	2008	PSC08‐06	JX680563
PSC08‐06‐AZ	29	Hero Inlet	PAL	−64°46′28.38″	−64°3′17.039″	0–35	2008	PSC08‐06	JX680564
PSC08‐06‐BA	29	Hero Inlet	PAL	−64°46′28.38″	−64°3′17.039″	0–35	2008	PSC08‐06	JX680565
PSC08‐06‐BD	29	Norsel Point	PAL	−64°45′38.279″	−64°5′52.44″	0–35	2008	PSC08‐06	JX680566
PSC08‐06‐BE	29	Norsel Point	PAL	−64°45′38.279″	−64°5′52.44″	0–35	2008	PSC08‐06	JX680567
PSC08‐06‐BF	29	Norsel Point	PAL	−64°45′38.279″	−64°5′52.44″	0–35	2008	PSC08‐06	JX680568
PSC08‐06‐BG	29	Stepping Stones Island	PAL	−64°47′11.4″	−63°59′49.801″	0–35	2008	PSC08‐06	JX680569
PSC08‐06‐BH	29	Stepping Stones Island	PAL	−64°47′11.4″	−63°59′49.801″	0–35	2008	PSC08‐06	JX680570
PSC08‐06‐BJ	29	Stepping Stones Island	PAL	−64°47′11.4″	−63°59′49.801″	0–35	2008	PSC08‐06	JX680571
PSC08‐06‐BK	29	Hero Inlet	PAL	−64°46′28.38″	−64°3′17.039″	0–35	2008	PSC08‐06	JX680572
PSC08‐06‐E	29	Janus Island	PAL	−64°47′6.421″	−64°6′7.499″	0–35	2008	PSC08‐06	JX680573
PSC08‐06‐F	29	Janus Island	PAL	−64°47′6.421″	−64°6′7.499″	0–35	2008	PSC08‐06	JX680574
PSC08‐06‐G	29	Janus Island	PAL	−64°47′6.421″	−64°6′7.499″	0–35	2008	PSC08‐06	JX680575
PSC08‐06‐K	29	Bonaparte Point	PAL	−64°46′39.72″	−64°3′59.159″	0–35	2008	PSC08‐06	JX680576
PSC08‐06‐L	29	Bonaparte Point	PAL	−64°46′39.72″	−64°3′59.159″	0–35	2008	PSC08‐06	JX680577
PSC08‐06‐M	29	Bonaparte Point	PAL	−64°46′39.72″	−64°3′59.159″	0–35	2008	PSC08‐06	JX680578
PSC08‐06‐Q	29	Bonaparte Point	PAL	−64°46′39.72″	−64°3′59.159″	0–35	2008	PSC08‐06	JX680579
WAMS103988	29	Ross Sea	RS	−77°34′18.12″	163°30′42.12″		2006	AMLR 2006	ON179525
G137_10_29	29	Bransfield Strait	BS	−63°23′3.001″	−60°3′24.001″	146	2004	LMG04‐14	EU823163A
USNM1120716	29	Bransfield Strait	BS	−63°23′3.001″	−60°3′24.001″	277	2004	LMG04‐14	EU823163
WAMS103989	29	Bransfield Strait	BS	−63°23′3.001″	−60°3′24.001″		2004	LMG04‐14	ON179526
USNM1120705	29	Bransfield Strait	BS	−63°23′3.001″	−60°3′24.001″	277	2004	LMG04‐14	EU823188
USNM1120713	29	Bransfield Strait	BS	−63°23′3.001″	−60°3′24.001″	277	2004	LMG04‐14	EU823189
USNM1120824	29	Bransfield Strait	BS	−63°23′3.001″	−60°3′24.001″	277	2004	LMG04‐14	EU823190
USNM1121291	29	Bransfield Strait	BS	−63°23′3.001″	−60°3′24.001″	277	2004	LMG04‐14	EU823163
USNM1120704	29	Bransfield Strait	BS	−63°23′3.001″	−60°3′24.001″	277	2004	LMG04‐14	EU823163
G137_10_29	29	Bransfield Strait	BS	−63°23′3.001″	−60°3′24.001″	277	2004	LMG04‐14	EU823163B
G137_6_29	29	Bransfield Strait	BS	−63°23′3.001″	−60°3′24.001″	277	2004	LMG04‐14	EU823191
G137_10_29	29	Bransfield Strait	BS	−63°23′3.001″	−60°3′24.001″	277	2004	LMG04‐14	EU823163C
G137_10_29	29	Bransfield Strait	BS	−63°23′3.001″	−60°3′24.001″	277	2004	LMG04‐14	EU823163D
G137_10_29	29	Bransfield Strait	BS	−63°23′3.001″	−60°3′24.001″	277	2004	LMG04‐14	EU823163E
USNM1121592	29	Bransfield Strait	BS	−63°8′50.28″	−57°7′26.461″	146	2006	LMG06‐05	EU823162
USNM1121596	29	Bransfield Strait	BS	−63°8′50.28″	−57°7′26.461″	146	2006	LMG06‐05	EU823162
USNM1121616	29	Bransfield Strait	BS	−63°8′50.28″	−57°7′26.461″	146	2006	LMG06‐05	EU823168
USNM1121581	29	Bransfield Strait	BS	−63°8′50.28″	−57°7′26.461″	146	2006	LMG06‐05	EU823170
USNM1121593	29	Bransfield Strait	BS	−62°49′0.3″	−56°39′28.62″	146	2006	LMG06‐05	EU823163
USNM1121606	29	Bransfield Strait	BS	−63°8′50.28″	−57°7′26.461″	146	2006	LMG06‐05	EU823164
USNM1122216	29	Bransfield Strait	BS	−62°32′43.199″	−55°21′57.06″	149	2006	AMLR 2006 ‐ Leg II	EU823146
USNM1121322	29	Bransfield Strait	BS	−62°32′43.199″	−55°21′57.06″	149	2006	AMLR 2006 ‐ Leg II	EU823214
USNM1121331	29	Bransfield Strait	BS	−62°32′43.199″	−55°21′57.06″	149	2006	AMLR 2006 ‐ Leg II	EU823147
USNM1121313	29	Bransfield Strait	BS	−62°32′43.199″	−55°21′57.06″	149	2006	AMLR 2006 ‐ Leg II	EU823149
USNM1121348	29	Bransfield Strait	BS	−63°0′1.321″	−58°5′0.6″	235	2006	AMLR 2006 ‐ Leg II	EU823215
USNM1120719	29	Bransfield Strait	BS	−62°49′0.3″	−56°39′28.62″	108	2006	AMLR 2006 ‐ Leg II	EU823146
USNM1120720	29	Bransfield Strait	BS	−62°36′46.681″	−56°36′36.9″	167	2006	AMLR 2006 ‐ Leg II	EU823159
ZSM20012286‐7	29	Bransfield Strait	BS	−63°4′41.999″	−57°31′36.001″	95	2000	EASIZ 3	EU823218
ZSM20012307‐1	29	Bransfield Strait	BS	−63°4′41.999″	−57°31′36.001″	95	2000	EASIZ 3	EU823159
ZSM20012307‐2	29	Bransfield Strait	BS	−63°4′41.999″	−57°31′36.001″	95	2000	EASIZ 3	EU823171
ZSM20012307‐3	29	Bransfield Strait	BS	−63°4′41.999″	−57°31′36.001″	95	2000	EASIZ 3	EU823172
ZSM20012307‐4	29	Bransfield Strait	BS	−63°4′41.999″	−57°31′36.001″	95	2000	EASIZ 3	EU823173
WAMS103992	29	Zappart Island	DS	−68°30′16.2″	78°4′59.88″	0–35	2010	Australian Antarctic Division	ON179527
WAMS103997	29	Janus Island	PAL	−64°47′6.421″	−64°6′7.499″		2010	PSC10‐10	ON179528
WAMS103502	29	Palmer Dock	PAL	−64°46′28.499″	−64°3′17.341″	18	2010	PSC10‐10	ON179529
WAMS103998	29	Litchfield Island	PAL	−64°46′27.12″	−64°5′13.621″	30	2010	PSC10‐10	ON179530
WAMS103503	29	Palmer Dock	PAL	−64°46′28.499″	−64°3′17.341″	18	2010	PSC10‐10	ON179531
WAMS102923	29	Norsel Point	PAL	−64°45′38.279″	−64°5′52.44″	27–35	2010	PSC10‐10	ON179532
WAMS102924	29	Stepping Stones	PAL	−64°47′11.4″	−63°59′49.801″		2010	PSC10‐10	ON179533
WAMS103549	29	Litchfield Island	PAL	−64°45′40.619″	−64°5′27.359″		2010	PSC10‐10	ON179534
WAMS103508	29	Hermit Island	PAL	−64°48′8.341″	−64°1′26.281″	15–36	2010	PSC10‐10	ON179535
WAMS103510	29	Lemaire Island	PAL	−65°4′37.081″	−63°58′10.859″		2010	PSC10‐10	ON179536
WAMS103509	29	Norsel Point	PAL	−64°45′38.279″	−64°5′52.44″	27	2010	PSC10‐10	ON179537
WAMS103556	29	Bahia Pariso	PAL	−64°46′49.62″	−64°5′44.999″		2010	PSC10‐10	ON179538
WAMS103552	29	Bonaparte Island	PAL	−64°46′39.72″	−64°3′59.159″		2010	PSC10‐10	ON179539
WAMS103929	29	Litchfield Island	PAL	−64°45′40.619″	−64°5′27.359″		2010	PSC10‐10	ON179540
WAMS103934	29	Janus Island	PAL	−64°47′6.421″	−64°6′7.499″		2010	PSC10‐10	ON179541
WAMS103937	29	Janus Island	PAL	−64°47′6.421″	−64°6′7.499″		2010	PSC10‐10	ON179542
WAMS103542	29	Norsel Point	PAL	−64°45′38.279″	−64°5′52.44″		2010	PSC10‐10	ON179543
WAMS103541	29	Stepping Stones	PAL	−64°47′11.4″	−63°59′49.801″		2010	PSC10‐10	ON179544
WAMS103540	29	Stepping Stones	PAL	−64°47′11.4″	−63°59′49.801″		2010	PSC10‐10	ON179545
WAMS103539	29	Stepping Stones	PAL	−64°47′11.4″	−63°59′49.801″		2010	PSC10‐10	ON179546
WAMS103538	29	Stepping Stones	PAL	−64°47′11.4″	−63°59′49.801″		2010	PSC10‐10	ON179547
WAMS103537	29	Stepping Stones	PAL	−64°47′11.4″	−63°59′49.801″	21	2010	PSC10‐10	ON179548
WAMS103939	29	Norsel Point	PAL	−64°45′40.619″	−64°5′27.359″	27–35	2010	PSC10‐10	ON179549
WAMS103940	29	Norsel Point	PAL	−64°45′40.619″	−64°5′27.359″	27–35	2010	PSC10‐10	ON179550
WAMS103942	29	Norsel Point	PAL	−64°45′40.619″	−64°5′27.359″	27–35	2010	PSC10‐10	ON179551
WAMS103944	29	Norsel Point	PAL	−64°45′38.279″	−64°5′52.44″		2010	PSC10‐10	ON179552
WAMS103530	29	Norsel Point	PAL	−64°45′38.279″	−64°5′52.44″	27–35	2010	PSC10‐10	ON179553
WAMS103945	29	Norsel Point	PAL	−64°45′38.279″	−64°5′52.44″	27–35	2010	PSC10‐10	ON179554
WAMS103529	29	Norsel Point	PAL	−64°45′38.279″	−64°5′52.44″	27–35	2010	PSC10‐10	ON179555
WAMS103947	29	Norsel Point	PAL	−64°45′38.279″	−64°5′52.44″	27–35	2010	PSC10‐10	ON179556
WAMS103948	29	Gamage Point	PAL	−64°46′28.56″	−64°3′24.901″	20–33	2010	PSC10‐10	ON179557
WAMS103526	29	Gamage Point	PAL	−64°46′28.56″	−64°3′24.901″	20–33	2010	PSC10‐10	ON179558
WAMS103958	29	Stepping Stones	PAL	−64°47′11.4″	−63°59′49.801″	30–36	2010	PSC10‐10	ON179559
WAMS103959	29	Stepping Stones	PAL	−64°47′11.4″	−63°59′49.801″	30–36	2010	PSC10‐10	ON179560
WAMS103968	29	Janus Island	PAL	−64°47′6.421″	−64°6′7.499″		2010	PSC10‐10	ON179561
WAMS103969	29	Janus Island	PAL	−64°46′39.72″	−64°3′59.159″		2010	PSC10‐10	ON179562
WAMS103979	29	Hero Inlet	PAL	−64°46′28.38″	−64°3′17.039″		2010	PSC10‐10	ON179563
SIOBICM12484	29	Bransfield Strait	BS	−62°51′54″	−57°12′39.6″	161–163	2011	NBP11_05	ON179564
SIOBICM12485	29	Bransfield Strait	BS	−62°51′54″	−57°12′39.6″	161–163	2011	NBP11_05	ON179565
SIOBICM12481	29	Bransfield Strait	BS	−62°51′54″	−57°12′39.6″	161–163	2011	NBP11_05	ON179566
SIOBICM12480	29	Bransfield Strait	BS	−62°51′54″	−57°12′39.6″	161–163	2011	NBP11_05	ON179567
SIOBICM12479	29	Bransfield Strait	BS	−62°51′54″	−57°12′39.6″	161–163	2011	NBP11_05	ON179568
SIOBICM12555	29	Bransfield Strait	BS	−62°52′10.236″	−57°13′0.552″	150–247	2011	NBP11_05	ON179569
SIOBICM12492	29	Bransfield Strait	BS	−62°51′54″	−57°12′39.6″	161–163	2011	NBP11_05	ON179570
SIOBICM12645	29	Bransfield Strait	BS	−62°51′54″	−57°12′39.6″	161–163	2011	NBP11_05	ON179571
SIOBICM12491	29	Bransfield Strait	BS	−62°51′54″	−57°12′39.6″	161–163	2011	NBP11_05	ON179572
SIOBICM12490	29	Bransfield Strait	BS	−62°51′54″	−57°12′39.6″	161–163	2011	NBP11_05	ON179573
SIOBICM12489	29	Bransfield Strait	BS	−62°51′54″	−57°12′39.6″	161–163	2011	NBP11_05	ON179574
SIOBICM12488	29	Bransfield Strait	BS	−62°51′54″	−57°12′39.6″	161–163	2011	NBP11_05	ON179575
SIOBICM12487	29	Bransfield Strait	BS	−62°51′54″	−57°12′39.6″	161–163	2011	NBP11_05	ON179576
SIOBICM12486	29	Bransfield Strait	BS	−62°51′54″	−57°12′39.6″	161–163	2011	NBP11_05	ON179577
SIOBICM12483	29	Bransfield Strait	BS	−62°51′54″	−57°12′39.6″	161–163	2011	NBP11_05	ON179578
SIOBICM12482	29	Bransfield Strait	BS	−62°51′54″	−57°12′39.6″	161–163	2011	NBP11_05	ON179579
SIOBICM12499A	29	Bransfield Strait	BS	−63°19′22.8″	−59°51′3.6″	197–199	2011	NBP11_05	ON179580
SIOBICM12194	29	Coronation Island	SOI	−60°45′5.04″	−44°11′55.32″	166	2009	AMLR 2009	ON179581
SIOBICM17143	29	Elephant Island	EI	−61°9′51.998″	−56°1′30″	150	2012	PS79	ON179582
SIOBICM17577	29	Bransfield Strait	BS	−62°51′53.64″	−57°12′38.52″	161–163	2011	NBP11‐05	ON179583
SIOBICM17579	29	Bransfield Strait	BS	−62°51′53.64″	−57°12′38.52″	161–163	2011	NBP11‐05	ON179584
WAMS103558	29	Low Island	SSI	−63°16′59.999″	−62°9′0″		2010	LMG‐SC10	ON179585
WAMS103562	29	Low Island	SSI	−63°16′59.999″	−62°9′0″		2010	LMG‐SC10	ON179586
WAMS103563	29	Low Island	SSI	−63°16′59.999″	−62°9′0″		2010	LMG‐SC10	ON179587
WAMS103564	29	Low Island	SSI	−63°16′59.999″	−62°9′0″		2010	LMG‐SC10	ON179588
WAMS103565	29	Low Island	SSI	−63°16′59.999″	−62°9′0″		2010	LMG‐SC10	ON179589
WAMS103566	29	Low Island	SSI	−63°16′59.999″	−62°9′0″		2010	LMG‐SC10	ON179590
WAMS103567	29	Low Island	SSI	−63°16′59.999″	−62°9′0″		2010	LMG‐SC10	ON179591
WAMS103573	29	Low Island	SSI	−63°16′59.999″	−62°9′0″		2010	LMG‐SC10	ON179592
SIOBICM17141	29	Elephant Island	EI	−61°9′32.4″	−56°1′19.2″	150	2012	PS79	ON179593
SIOBICM17142	29	Elephant Island	EI	−61°9′32.4″	−56°1′19.2″	150	2012	PS79	ON179594
SIOBICM17314	29	Elephant Island	EI	−61°2′34.8″	−55°45′28.8″	130	2012	PS79	ON179595
SIOBICM17360	29	Elephant Island	EI	−61°2′34.8″	−55°45′28.8″	130	2012	PS79	ON179596
WAMS103651	29	Allan's Wall	PAL	−64°45′41.879″	−64°5′29.159″	39	2018	PSC_2018	ON179597
SIOBICM17139	29	Elephant Island	EI	−61°9′32.4″	−56°1′19.2″	150	2012	PS79	ON179598
WAMS104002	29	Low Island	SSI	−63°16′59.999″	−62°9′0″		2010	LMG‐SC10	ON179599
WAMS104003	29	Low Island	SSI	−63°16′59.999″	−62°9′0″		2010	LMG‐SC10	ON179600
SIOBICM17551	42	South Georgia	SG	−53°48′0″	−37°13′8.4″	143–145	2011	NBP11‐05	ON179698
SIOBICM17549	42	South Georgia	SG	−53°48′0″	−37°13′8.4″	143–145	2011	NBP11_05	ON179699
SIOBICM12891	42	South Georgia	SG	−53°48′0″	−37°13′8.4″	143–145	2011	NBP11_05	ON179700
SIOBICM17531	42	Shag Rocks	SR	−53°34′30″	−41°40′44.4″	134–136	2011	NBP11_05	ON179701
SIOBICM12546	42	South Georgia	SG	−53°48′0″	−37°13′8.4″	143–145	2011	NBP11_05	ON179702
SIOBICM12551	42	South Georgia	SG	−53°46′1.2″	−37°13′1.2″	143–151	2011	NBP11_05	ON179703
SIOBICM12550	42	South Georgia	SG	−53°46′1.2″	−37°13′1.2″	143–151	2011	NBP11_05	ON179704
SIOBICM13167	42	South Georgia	SG	−53°38′52.8″	−37°16′26.4″	139–140	2013	NBP13_03	ON179705
SIOBICM13238	42	South Georgia	SG	−53°38′52.8″	−37°16′26.4″	139–140	2013	NBP13_03	ON179706
SIOBICM13204	42	South Georgia	SG	−53°38′52.8″	−37°16′26.4″	139–140	2013	NBP13_03	ON179707
SIOBICM13213	42	South Georgia	SG	−53°40′37.2″	−37°14′45.6″	137	2013	NBP13_03	ON179708
SIOBICM18258	42	Shag Rocks	SR	−53°32′56.4″	−41°38′6″	127–128	2013	NBP13_03	ON179709
SIOBICM13219	42	Shag Rocks	SR	−53°31′51.6″	−41°37′19.2″	126–127	2013	NBP13_03	ON179710
SIOBICM13199	42	Shag Rocks	SR	−53°32′34.8″	−41°37′33.6″	127–130	2013	NBP13_03	ON179711
SIOBICM18293	42	Burdwood Bank	BB	−53°53′31.2″	−60°40′51.6″	133–134	2013	NBP13_03	ON179712

Abbreviation: COI, cytochrome oxidase I.
